# Impact of industrial phosphate waste discharge on the marine environment in the Gulf of Gabes (Tunisia)

**DOI:** 10.1371/journal.pone.0197731

**Published:** 2018-05-17

**Authors:** Akram El Kateb, Claudio Stalder, Andres Rüggeberg, Christoph Neururer, Jorge E. Spangenberg, Silvia Spezzaferri

**Affiliations:** 1 Department of Geosciences, University of Fribourg, Chemin du Musée 6, Fribourg, Switzerland; 2 Federal Office of Public Health FOPH, Radiation Protection Division, Schwarzenburgstrasse 157, Bern, Switzerland; 3 Institute of Earth Surface Dynamics (IDYST), University of Lausanne, Lausanne, Switzerland; Universidade de Aveiro, PORTUGAL

## Abstract

The marine environment in the Gulf of Gabes (southern Tunisia) is severely impacted by phosphate industries. Nowadays, three localities, Sfax, Skhira and Gabes produce phosphoric acid along the coasts of this Gulf and generate a large amount of phosphogypsum as a waste product. The Gabes phosphate industry is the major cause of pollution in the Gulf because most of the waste is directly discharged into the sea without preliminary treatment. This study investigates the marine environment in the proximity of the phosphate industries of Gabes and the coastal marine environment on the eastern coast of Djerba, without phosphate industry. This site can be considered as "pristine" and enables a direct comparison between polluted and “clean” adjacent areas.

Phosphorous, by sequential extractions (SEDEX), Rock-Eval, C, H, N elemental analysis, and stable carbon isotope composition of sedimentary organic matter, X-ray diffraction (qualitative and quantitative analysis) were measured on sediments. Temperature, pH and dissolved oxygen were measured on the water close to the sea floor of each station to estimate environmental conditions. These analyses are coupled with video surveys of the sea floor. This study reveals clear differentiations in pollution and eutrophication in the investigated areas.

## Introduction

Phosphorus plays a major role in several biological processes like energy transfer and is present e.g., in genetic material and in bones [1–3]. It is also an important element of fertilizers in agriculture production. Two forms of crystallized phosphorus can be mainly found: “white phosphorus” with a tetrahedral structure, “red phosphorus” which is present under polymeric forms and mineral forms such as phosphate [4].

Phosphate deposits in North Africa and in the Middle East have sedimentary origin [5]. Phosphate rocks (phosphorite) in Tunisia formed during Palaeocene-Eocene time in the Gafsa basin in western part of Tunisia, around the Kasserine and Djeffara Islands and the Algerian promontory [6–11]. Phosphorites in the Gafsa basin contain authigenic apatite from pellets and coprolites, phosphatized fossils, coated grains and oolites and biogenic apatite fossils such as fish teeth and skeletal fragments [9].

Phosphorites are chemically treated to produce phosphoric acid. This process generates a large volume of phosphogypsum (PG), around 5 tons per ton of phosphoric acid [12]. Phosphogypsum contains several pollutants like heavy metals, fluorine, phosphorus and even radionuclides generating radioactivity [12–18]. In 2010, Tunisia was a leading country of phosphate production (8 million tons) being the fifth phosphate producer worldwide with around 13 million tons of phosphate ore extracted. Today the country treats 80% of his phosphorite production in four main industries. Three of them are located along the coast of the Gulf of Gabes. At Sfax and Skhira, PG is stored in large stacks around the industry complex.

The large industrial site at Gabes has several units of phosphorite treatment and the totality of the waste (PG, industrial sludge, waste water) is directly discharged into the sea through a canal [19] with relevant environmental impact [13, 14, 20, 21, 22, 23].

Consequence of the large production of PG is a severe heavy metal contamination of seawater and sediments (e.g., [24–28]), which has a strong impact on the marine fauna by bioaccumulation effect since elementary phosphorus can be assimilated in sediments in various phases [29–31].

The phosphorus sequential extraction (SEDEX) from sediments allows quantifying five sedimentary phosphorus reservoirs [29, 31] and provides important information about the phosphorus cycle also in context of polluted sites [2, 32, 33, 34, 35]. This study investigates the effect of large quantity of PG wastes discharged into the Gulf of Gabes, the role of the 5 phosphorus phases of Ruttenberg et al. [31] in modifying environmental conditions and compares the Gulf with an analogue coastal marine environment (eastern coast of Djerba), which has no phosphate production industry and can consider as “pristine”.

As a nutrient, phosphorus has the potential to trigger phytoplankton blooms [36], thus to increase organic matter (OM) input to the seafloor and consequently to trigger eutrophication [37]. Therefore, this study also investigates the origin of OM to identify its origin and the possible link with pollutants.

## Materials and methods

### Ethic statement

No specific permissions were required to collect samples on the entire working area for this study. All the locations where the samples were collected are public access. In addition, the field studies did not involve endangered or protected species.

### Study site and sampling strategy

The Gulf of Gabes is approximately 90 km wide, 100 km long and bounded by the Kerkhenna Islands to the north and the Djerba Island to the south. It presents some unique characteristics in the Mediterranean Sea such as a gently sloping bathymetry to a water depth of 50 m at around 130 km from the coastline and tidal amplitude, which is the highest in the Mediterranean Sea, exceeding 1.7 m [38].

The Gulf of Gabes plays an important role in Tunisian economy as a well-known fishing reserve. In the past 25 years, 65% of Tunisian fishery was from this region [39]. At the beginning of the 20th century, the majority of its seafloor was colonized by seagrass. *Posidonia oceanica* was the dominant marine plant in the region providing an ideal nursery environment for many species [39, 40].

**Fig 1:**
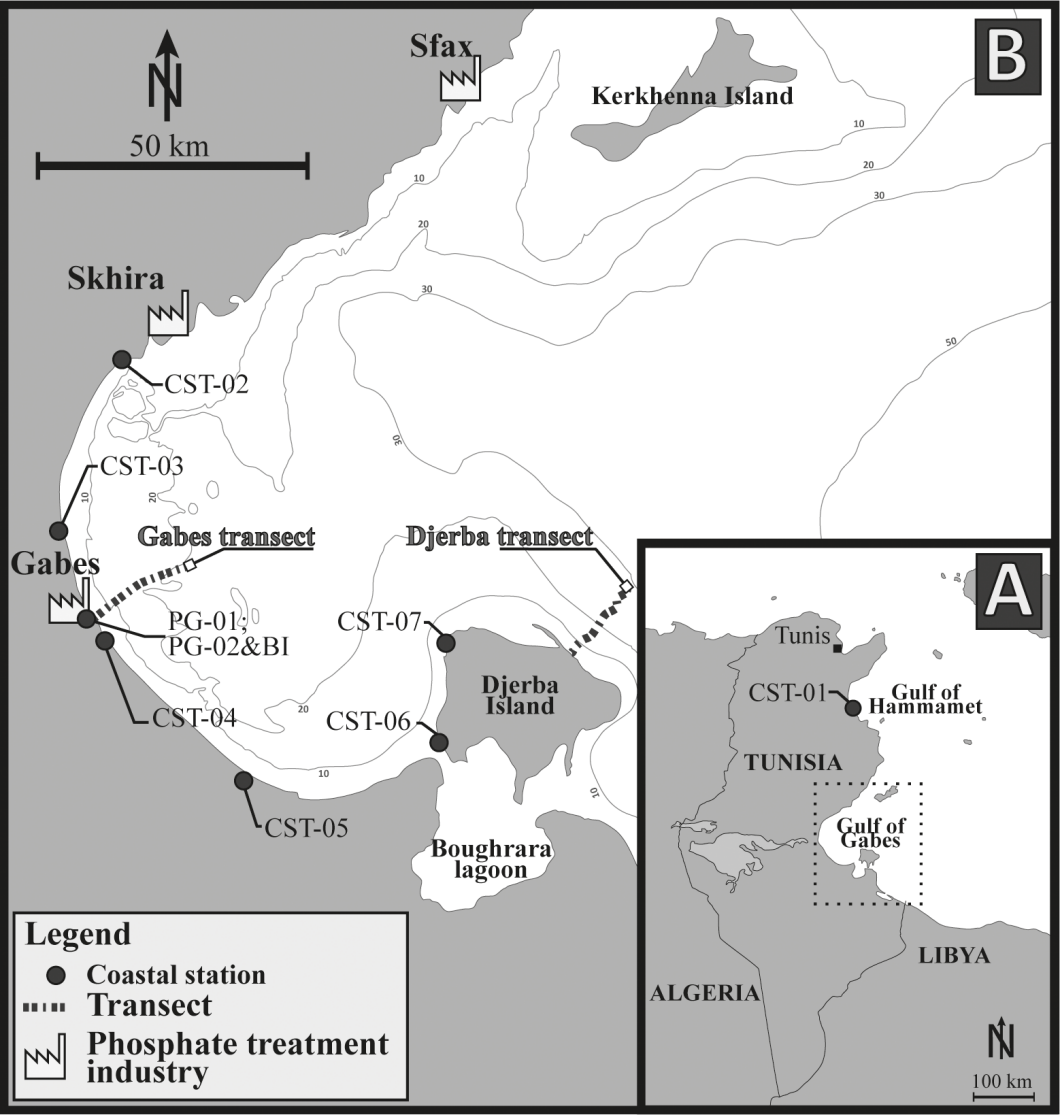
Maps of the study area showing the sampling sites. (A) Maps of Tunisia showing the location of the coastal station CST-01. (B) Localities of the Gabes and, Djerba transects and other coastal stations inside the Gulf of Gabes. This figure is similar but not identical to the original image, and is therefore for illustrative purpose only.

Seven coastal stations were sampled at >1 m water depth. They encompass over 200 km along the eastern Tunisian coast from the Gulf of Hammamet to the northern edge of Djerba Island but most of the stations are located along the Gulf of Gabes (Fig 1).

Two transects were sampled perpendicularly to the coast. The Gabes transect was sampled between the industrial and the fishing harbours, it includes 16 stations from the phosphate industries to offshore. Samples were collected approximately every 1 km from the coast to 17.3 km offshore. (Fig 1). The first station GBS-01 is located 400 m from the shoreline and 800 m from the waste industry discharge area. Water depth ranges from 4.5 m (GBS-01) to 19.5 m (GBS-10, GBS-11 and GBS-15). The Djerba transect includes 15 stations and is located on the eastern coast of Djerba Island (Fig 1). Samples were collected along a 13.8 km long transect with the first station DJB-01 at 600 m distance from the coast. Water depth ranges from 5.1 m (DJB-01) to 26.8 m (DJB-15) (Fig 1).

Three additional samples were collected from the waste discharge canal of the phosphate industries of Gabes. Two of them contain PG (PG-01 and PG-02) and were collected in different periods (PG-01 in January 2014 and PG-02 in July 2014). The BI sample, composed of dark industrial sludge, was collected in July 2014. Geographical coordinates of transects and coastal stations are in Table 1.

**Table 1:** Geographical coordinates of stations of Gabes and Djerba transects and coastal stations.

**Gabes transect**		**Djerba transect**		**Costal stations**	
**Sample**	**GPS coordinates**	**Sample**	**GPS coordinates**	**Sample**	**GPS coordinates**
**GBS-01**	N 33°54'36.66" / E 10° 6'34.74"	**DJB-01**	N 33°52'13.26" / E 10°58'22.02"	**CST-01**	N 35°53'49.15" / E 10°35'45.79"
**GBS-02**	N 33°54'52.32" / E 10° 7'11.76"	**DJB-02**	N 33°52'33.12" / E 10°59'1.86"	**CST-02**	N 34°17'19.98" / E 10° 5'45.00"
**GBS-03**	N 33°55'9.54" / E 10° 7'44.10"	**DJB-03**	N 33°52'49.38" / E 10°59'24.60"	**CST-03**	N 34° 2'15.84" / E 10° 2'9.36"
**GBS-04**	N 33°55'29.82" / E 10° 8'15.36"	**DJB-04**	N 33°53'3.84" / E 10°59'57.84"	**CST-04**	N 33°53'5.76" / E 10° 7'13.92"
**GBS-05**	N 33°55'48.00" / E 10° 8'46.92"	**DJB-05**	N 33°53'32.82" / E 11° 0'15.30"	**CST-05**	N 33°41'57.90" / E 10°21'34.02"
**GBS-06**	N 33°56'7.74" / E 10° 9'15.72"	**DJB-06**	N 33°53'53.34" / E 11° 0'31.20"	**CST-06**	N 33°43'39.00" / E 10°44'22.50"
**GBS-07**	N 33°56'30.60" / E 10° 9'46.74"	**DJB-07**	N 33°54'9.78" / E 11° 0'58.38"	**CST-07**	N 33°51'34.62" / E 10°44'41.40"
**GBS-08**	N 33°56'57.24" / E 10°10'18.84"	**DJB-08**	N 33°54'45.54" / E 11° 1'21.84"		
**GBS-09**	N 33°57'16.20" / E 10°10'53.82"	**DJB-09**	N 33°55'9.42" / E 11° 1'48.78"	**Waste industries**	
**GBS-10**	N 33°57'29.34" / E 10°11'36.24"	**DJB-10**	N 33°55'21.90" / E 11° 2'24.12"	**Sample**	**GPS coordinates**
**GBS-11**	N 33°57'40.08" / E 10°12'9.30"	**DJB-11**	N 33°55'48.36" / E 11° 3'4.68"	**PG-01; PG-02 & BI**	N 33°54'46.62" / E 10° 5'53.82"
**GBS-12**	N 33°57'56.64" / E 10°12'47.04"	**DJB-12**	N 33°56'24.12" / E 11° 2'59.76"		
**GBS-13**	N 33°58'9.66" / E 10°13'22.20"	**DJB-13**	N 33°56'53.16" / E 11° 3'36.36"		
**GBS-14**	N 33°58'14.04" / E 10°13'58.86"	**DJB-14**	N 33°57'5.64" / E 11° 4'1.80"		
**GBS-15**	N 33°58'27.18" / E 10°14'39.18"	**DJB-15**	N 33°57'30.36" / E 11° 4'9.84"		
**GBS-16**	N 33°58'53.16" / E 10°16'15.60"				

### Sampling methods and in-situ data collection

Surface sediment samples (first centimetre) were collected for geochemical, sedimentological and mineralogical analyses. An Ekman-Birge box core (15x15x30 cm) was deployed at each station of both transects (Fig 1) to collect surface sediments. Sediments were photographed and described to document sedimentary facies. Around 15 cm^3^ of the first centimetre of surface sediment were collected in a falcon tube using a polyethylene spatula and all samples were kept at 4°C. Approximately 50 cm^3^ of surface sediment were subsampled at each station for grain size analysis.

The following data were collected in-situ along the two transects: A video survey of the seafloor was performed on each station to have an overview of the environment (e.g., marine flora and fauna, sedimentary facies). Videos were obtained by a GoPro® camera and a waterproof torch fixed on an aluminium frame. Water depth was measured with a Compass® echo sounder system. Water temperature, pH and dissolved oxygen were measured at the seafloor at each station. Water temperatures were measured using a Campbell Scientific® 107 temperature sensor with an accuracy of ±0.2°C at a range of -0°C to 50°C. pH was measured with a Campbell Scientific® ISFET CS525 probe, which can operates down to 70 m water depth. Temperature values are compensated with an accuracy of ±0.1 pH units at a temperature range to 0–40°C. Dissolved oxygen (DO) was measured with a Campbell Scientific® CS512 probe ranging from 0 to 50 mg/L and operating between 0 and 40°C at a pressure up to 2 bars with an accuracy of ±0.2 mg/L. Sensors were attached to an aluminium frame equipped with a tripod allowing the stability of system at the seafloor. A three-point calibration was performed for the pH (pH at 4.01, 7.00 and 10.00), the DO was calibrated based on the atmospheric pressure at sea level before sampling at each transect. Measurements were recorded on a datalogger CR1000 (Campbell Scientific®).

### Geochemical analyses

The SEDEX phosphorus extraction was performed on all sediment samples. They were dried at room temperature and manually grounded in an agate mortar to homogenize the sediment and to obtain a fine powder. They were successively dry sieved trough a 125 μm mesh to remove coarse particles, only the fine sediment fraction (<125 μm) was analysed. The phosphorus sequential extraction of Ruttenberg et al. [31], was performed on 80 mg of dry sediment to extract five phosphorus phases: 1) Exchangeable or loosely sorbed phosphorus (P_ex_); 2) Fe-bound phosphorus (P_Fe_); 3) Authigenic apatite: carbonate fluorapatite (CFA) + biogenic apatite + CaCO_3_-bound phosphorus (P_authi_); 4) Detrital apatite + other inorganic phosphorus (P_detr_); 5) Organic phosphorus (P_org_). The extraction was performed at the University of Fribourg and extracted phases were measured with a Bio-Tek Uvikon XS spectrophotometer using the molybdate blue method. The Fe-bound phosphorus (P_Fe_) phase and the Iron content were measured with an ICP-OES spectrometer Optima 7000 DV.

Rock-Eval analyses were performed on surface sediment samples following Rock-Eval6 technology [41] to obtain Total Organic Carbon (TOC in *wt*.%), Hydrogen Index (HI), Oxygen Index (OI), S1, S2 and S3 peaks, maximal temperature (T_max_) and mineral carbon (MINC) values. This technique uses temperature programmed heating of rocky sample under inert condition (anoxic). HI corresponds to the free hydrocarbons present in the sample (mg HC/g TOC) and is measured on S1 peak. OI represents the amount of hydrocarbons and compounds containing oxygen that are produced during the thermal cracking of kerogen (mg CO_2_/g TOC) and is determined from the S2 and S3 peaks. Maximal temperature is measured at the maximum of S2 peak. In addition, carbon oxidation is performed at high temperature (up to 850°C), which allows MINC determination [42, 43].

Total carbon, hydrogen and nitrogen (C,H,N) content (in *wt*.%) were measured in all surface sediment samples using a Thermo Finnigan Flash EA 1112 gas chromatography analyser. Rock-Eval and C, H, N analysis were performed on approximately 100 mg bulk sediment at the University of Lausanne.

Matrix effects, corresponding to carbon adsorption capacity of the sediment, can be quantified for a group of samples based on Rock-Eval data set. The positive intercepts of the regression line for a group of samples on S2 vs. TOC diagram indicate a matrix effect and the position of the intercept is the measure of the amount of absorption (g of OM absorbed by 1g of sediment) [44]. Matrix effect was quantified for samples from the Gabes and Djerba transects and coastal stations.

Different graphs can be used to qualify the type of the kerogen present in OM and determine its origin. For the present study, the S2 (mg/g) vs. TOC graph was used to determine if a matrix effect influences the measurements [44, 45]; the HI vs. T_max_ plot was used to determine the origin of the sedimentary organic matter [46].

Molar C/N ratio is calculated for sediment samples, C was determined from the TOC content in the sediment and N from the total nitrogen content (TN). Total organic carbon and TN values are converted into μmol/g to obtain the molar C/N ratio. However, for several samples (CST-01; CST-04; CST-05 and PG-01; PG-02) TN values were below the detection limit. Molar C/P is calculated for sediment samples, C was determined from the TOC content and P from P_org_ phase of SEDEX extraction.

Total phosphorus (P_total_) represents the sum of all phosphorus reservoirs (P_total_ = P_ex_ + P_Fe_ + P_authi_ + P_detr_ + P_org_). Proportion of phosphorus reservoirs (P_ex_; P_Fe_; P_authi_; P_detr_; P_org_) is calculated in percentage depending of the total phosphorus (P_total_) in sediment.

Stable carbon isotope composition of the sedimentary OM (*δ*^13^C_OM_ values) was measured on surface sediment samples with sufficient material for this analysis. Carbonate minerals were dissolved using a 10% HCl solution at 50°C and rinsed with milli-Q water, the process was repeated twice. Stable carbon isotopes composition was measured at the Stable Isotopes Laboratory (University of Lausanne) by flash combustion on a Carlo Erba 1108 elemental analyser (EA) connected to a Thermo Fisher Scientific Delta V IRMS that was operated in a continuous helium flow mode via a Conflo III split interface. Results were expressed as *δ*^13^C as the per mil (‰) deviations of the ratio of the heavy to light isotopes (^13^C/^12^C) relative to Vienna Pee Dee Belemnite (VPDB) standard. Reproducibility and accuracy were better than ± 0.1‰.

### Sedimentary, mineralogical and petrographic analyses

Grain size analysis was performed on all samples except the industrial waste samples (PG-01, PG-02 and BI) and GBS-03 because of their small volume. Sediments were wet sieved through four mesh sieves: 500 μm, 250 μm, 125 μm and 63 μm. The grain size distribution was obtained by weighing the size fractions and is expressed in percentage over the total dry sediment weight.

Sediments from both transects were analysed by X-ray powder diffraction (XRD) using a Powder X-ray diffractometer Rigaku Ultima IV with a cupper anode. Samples were powdered with an agate mortar to obtain a fine powder with a grain size between 5 and 15 μm. The measurements were operated at 40 kV and 40 mA using a 1-D DTEX detector with Bragg Brentano Optics at step size of 0.02°. The data were collected in the 5°-120° 2ɵ angular range. X-ray pattern treatment was carried out with Panalytical X’pert HighScore Plus to identify the mineralogical phases. A Rietveld refinement was performed, with the same software, to quantify mineralogical phases.

Five polished thin sections were obtained from the carbonate nodules and were analysed under a polarized microscope. Three thin sections were made on nodules collected at station GBS-03 (GBS-03A to C) and two at station GBS-04 (GBS-04A and GBS-04B).

## Results

### Water parameters

Water parameters measured along the two transects are presented in Table 2.

**Table 2:** Bottom water parameters from stations of Gabes and Djerba transects.

Gabes transect						Djerba transect					
Station	Distance from the coastline	Water depth	Water temperature	pH	Dissolved oxygen	Station	Distance from the coastline	Water depth	Water temperature	pH	Dissolved oxygen
**GBS-01**	0.4	4.5	28.1	6.2	7.6	**DJB-01**	0.6	5.1	26.7	7.7	9.1
**GBS-02**	1.5	7.3	28.6	5.7	6.1	**DJB-02**	1.7	6.4	26.4	7.7	9.3
**GBS-03**	2.4	9.6	27.4	7.8	7.7	**DJB-03**	2.5	8.7	26.3	7.7	9.0
**GBS-04**	3.5	9.1	27.3	7.8	7.8	**DJB-04**	3.5	10.7	26.2	7.7	8.8
**GBS-05**	4.4	12	27.3	7.9	7.8	**DJB-05**	4.4	12.7	26.2	7.7	8.8
**GBS-06**	5.4	12.9	27.5	7.8	6.9	**DJB-06**	5.1	12.2	26.1	7.7	8.9
**GBS-07**	6.4	14.4	27.3	7.9	7.4	**DJB-07**	6.0	14	26.1	7.8	9.2
**GBS-08**	7.6	15	27.3	7.9	7.5	**DJB-08**	7.1	17.2	25.9	7.8	8.8
**GBS-09**	8.7	18	27.3	7.9	7.1	**DJB-09**	8.2	17.6	25.8	7.8	9.0
**GBS-10**	9.8	19.5	27.4	7.9	7.3	**DJB-10**	9.1	21.3	25.3	7.6	8.7
**GBS-11**	10.7	19.5	27.4	7.9	7.2	**DJB-11**	10.4	22	25.6	7.9	8.0
**GBS-12**	11.8	15.4	27.4	8.0	7.6	**DJB-12**	11.1	24	25.6	7.9	8.0
**GBS-13**	12.7	19.5	27.4	8.0	7.5	**DJB-13**	12.4	25.3	25.6	7.9	8.0
**GBS-14**	13.6	17.6	27.4	8.0	7.1	**DJB-14**	13.1	26	25.6	7.9	8.0
**GBS-15**	14.7	19.5	27.4	8.0	7.4	**DJB-15**	13.8	26.8	25.6	7.9	8.0
**GBS-16**	17.3	18	27.2	8.0	6						

Distance from the coastline (km), water depth (m), water temperature (°C), pH and dissolved oxygen (mg/L) at the seafloor.

#### Gabes transect

**Fig 2:**
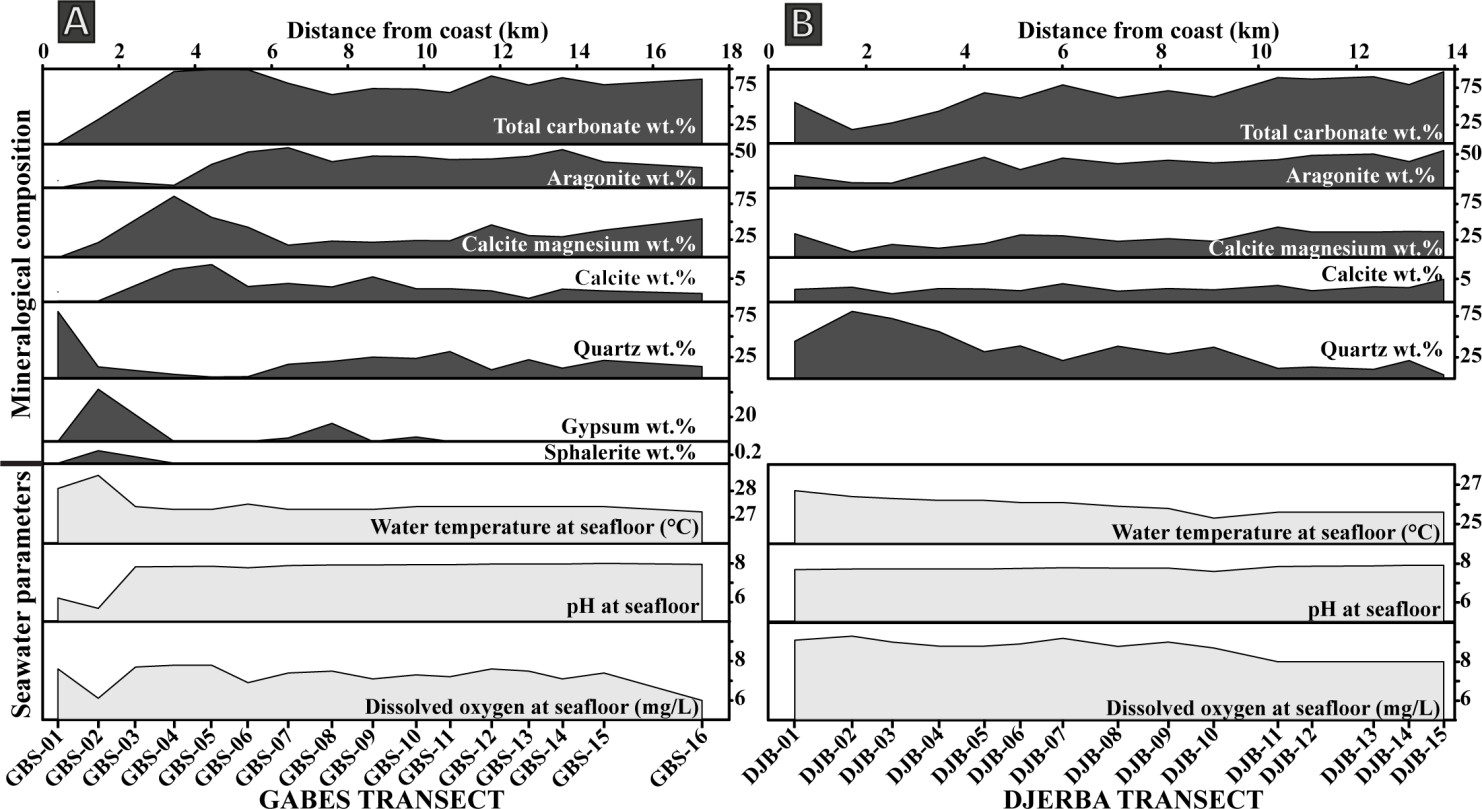
Variation in mineralogical composition and seawater parameters. (A) Gabes Transect. (B) Djerba transect.

Dissolved Oxygen (DO) values at the seafloor along the Gabes transect fluctuate from 7.1 to 7.8 mg/L in most of the stations. Only exceptions are values of 6.9 mg/L (Station GBS-05) and 6.1 and 6 mg/L (GBS-02 and GBS-16, respectively) (Fig 2). Bottom water temperatures along the Gabes transect fluctuate around 27.3°C, except for stations GBS-01 and GBS-02 where water temperatures exceed 28°C (Fig 2). The pH varies from 7.8 and 8.0. Remarkably lower pH values were measured at stations GBS-01 (6.2) and GBS-02 (5.7).

#### Djerba transect

**Fig 3:**
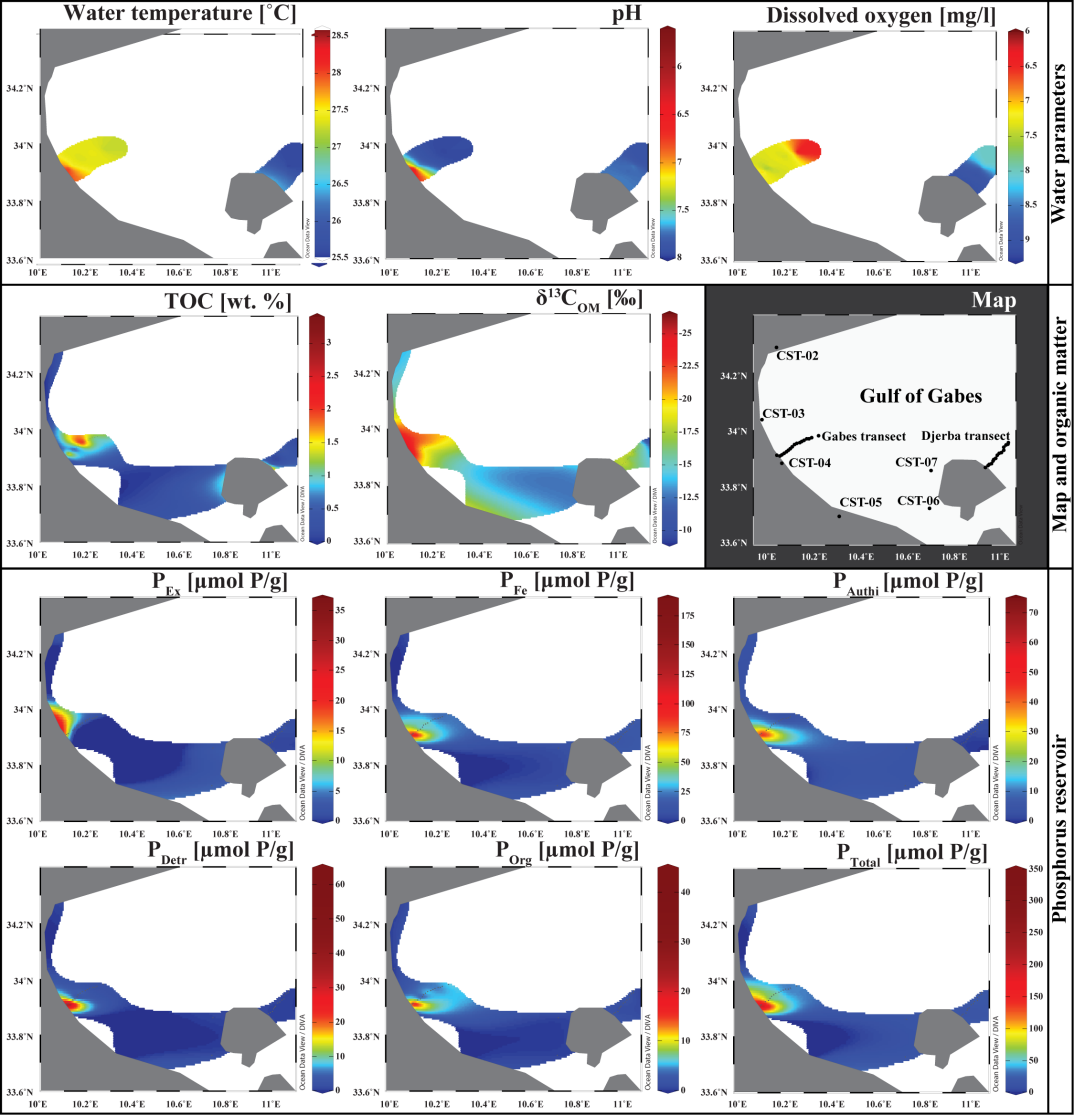
Isosurface in the Gulf of Gabes. Water parameters, TOC, carbon isotope composition of sedimentary organic matter, phosphorus reservoir concentrations are plot on a 2D Maps of the Gulf of Gabes. The maps are drawn by using the Ocean Data View software [47].

Bottom water parameters along the Djerba transect are significantly different from those of the Gabes (Table 2; Figs 2 and 3). In particular, they decrease with decreasing water depth from 26.7°C to 25.6°C. pH values increase from 7.7 at station DJB-01 to 7.9 at the more distal parts. Dissolved oxygen reaches a maximum of 9.1 mg/L at station DJB-01 and values remain high exceeding 9 mg/L along the first 2.5 kilometres (until DJB-03) (Fig 2). From station DJB-04 to station DJB-10, DO fluctuates from 8.7 to 9.2 mg/L. In the remaining stations DO decreases to a minimum of 8.0 mg/L (Fig 2).

### Sediment facies, mineralogy and nodule petrography

**Fig 4:**
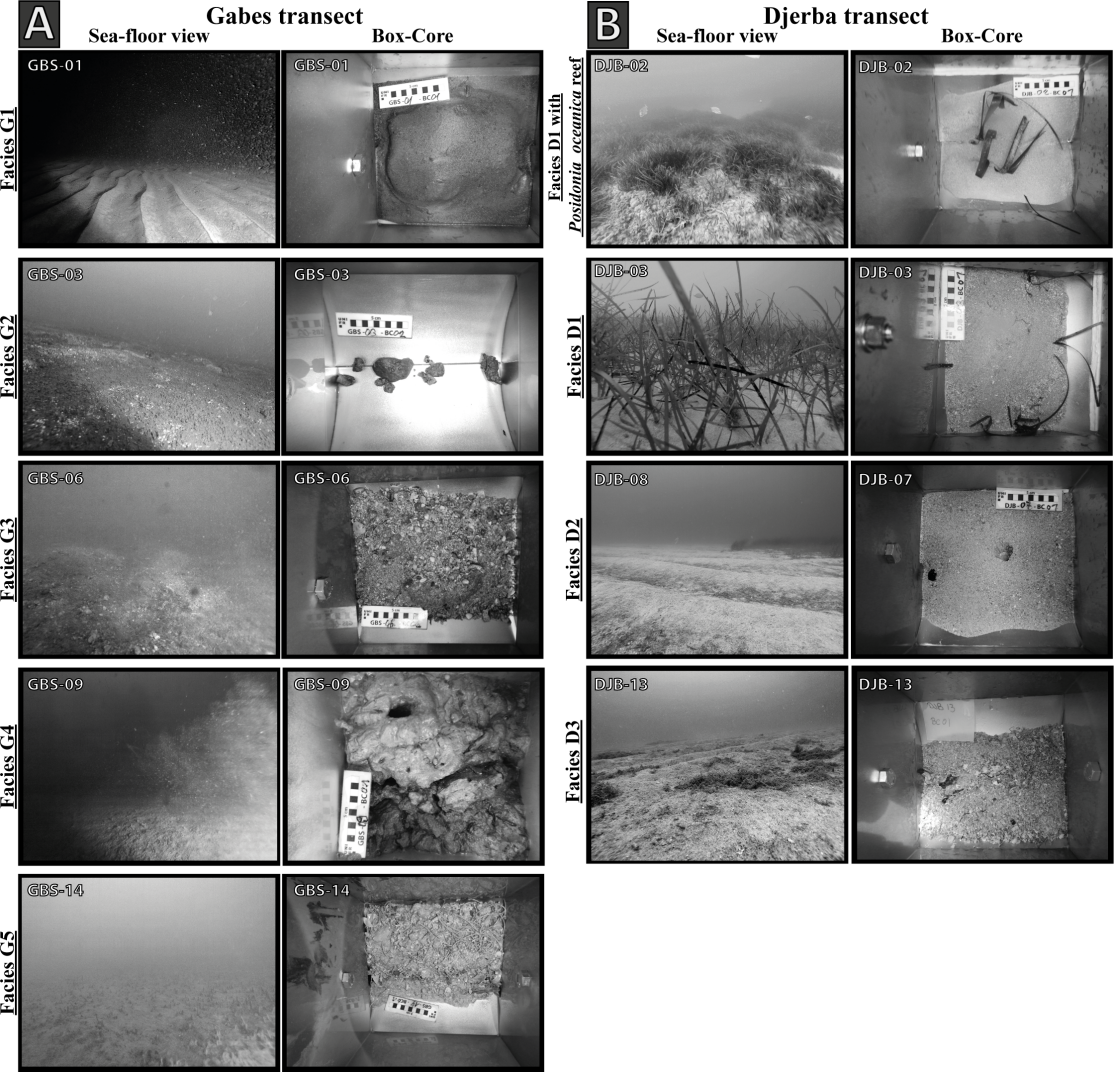
Illustration of the sedimentary facies. Images of seafloor and relative Box-Cores representing the sedimentary facies of (A) the Gabes and (B) the Djerba transects.

**Fig 5:**
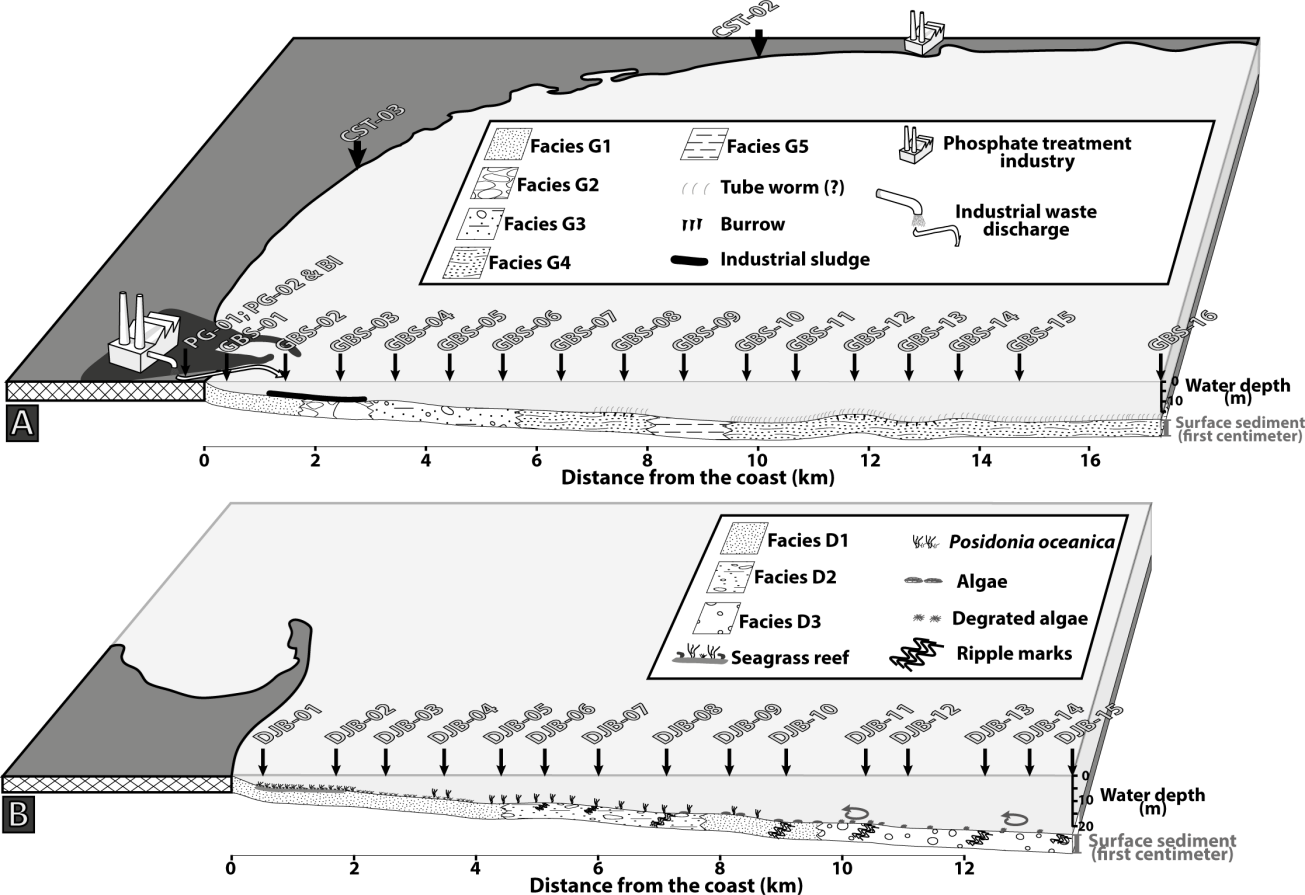
Sediment distribution. (A) Gabes transect. (B) Djerba transect.

Along the Gabes transect, five sedimentary facies are identified (Figs 4 and 5):

***Facies G1*** is present at stations GBS-01 and GBS-02 and is characterized by high content of siliciclastic grains (e.g. quartz).

***Facies G2*** occurs only at station GBS-03. Nodules of few centimetres diameters cover the seafloor forming a relatively hard substratum. The nodules are concretions of biogenic fragments (e.g. bivalve, bryozoan, foraminifera and coral) partially dissolved.

***Facies G3*** is present at stations GBS-04 to GBS-06 and is characterized by carbonate sand and biogenic fragments (bryozoans, bivalves, gastropods and coral).

***Facies G4*** is present at station GBS-07 and GBS-08, GBS-10 to GBS-16 and is characterized by a mix of large biogenic fragments (up to few centimetres) and fine sediment (clay).

***Facies G5*** is only present at station GBS-09 and is characterized by very fine sediments (clay and silt) and low content of biogenic fragments.

Along the Djerba transect three main sedimentary facies are identified (Figs 4 and 5):

***Facies D1*** is present from station DJB-01 to DJB-05 and at stations DJB-09 and DJB-10. Fine siliciclastic grains (quartz) mainly characterize this facies.

***Facies D2*** occurs at stations DJB-06 to DJB-08. It consists of a mix of sand and small (few millimetres) rounded biogenic fragments. Preservation state and shape of the biogenic fragments is poor due to mechanic erosion.

***Facies D3*** is present from station DJB-11 to DJB-15 and is composed by relatively well-preserved biogenic fragments of bivalves, calcareous algae, gastropods and bryozoans.

The seafloor at stations DBJ-01 and DJB-02 is densely covered by *P*. *oceanica* forming a seagrass barrier (Figs 4 and 5). Rhizomes of the sea-grass trap sediment forming small mounds or barrier reef meadow of *P*. *oceanica* (e.g. [48]). Ripple marks are observed at several stations (Fig 5) and are generally well developed (30 cm high).

All XRD analysis and Rietveld refinement results are presented in the Table 3. The main minerals present is surface sediment along the Gabes transect are aragonite, calcite, Mg-calcite and quartz (Fig 2). Additional minerals like orthoclase, halite, gypsum, sphalerite and kaolinite are also present, whereas calcite is relatively scarce. This mineral is absent at stations GBS-01 and GBS-02, while stations GBS-04 and GBS-05 have highest calcite contents of 7.1 and 8.2 *wt*.%, respectively. From station GBS-06 to GBS-16, the calcite content is relatively constant with an average of 2.8 *wt*.%. Mg-calcite reaches maximum value of 88.6 *wt*.% at GBS-04 station and it is absent at station GBS-01. It is abundant between GBS-02 to GBS-05 station but decreases between GBS-06 to GBS-16 (average is 39 *wt*.%). Aragonite is also absent in sample GBS-01 and relatively scarce in samples GBS-02 and GBS-03 with 11.1 *wt*.% and 3.9 *wt*.%, respectively. Quartz is very abundant at station GBS-01 with 80.5 *wt*.% but the quartz content fall to 3.7 *wt*.% at station GBS-04. From station GBS-07 to GBS-16, quartz content varies between 9.5 and 31.7 *wt*.%. Orthoclase was identified only at station GBS-01 with 18.3 *wt*.%. Gypsum was identified at several stations: GBS-02, GBS-07, GBS-08 to -10 and GBS-13 and is especially abundant at station GBS-02 with 42.6 *wt*.%. Sphalerite was detected only at the station GBS-02 with 0.3 *wt*.% and kaolinite with only 1.1 *wt*.% at station GBS-09.

**Table 3:** Mineralogical composition of surface sediment from Gabes and Djerba transects.

Sample Number	Total carbonate [wt.%]	Aragonite [wt.%]	Calcite magnesian [wt.%]	Calcite [wt.%]	Quartz [wt.%]	Halite [wt.%]	Gypsum [wt.%]	Orthoclase [wt.%]	Sphalerite [wt.%]	Kaolinite [wt.%]
**GBS-01**	0	0	0	0.0	80.5	1.2	0	18.3	0	0
**GBS-02**	32.0	11.1	20.9	0.0	12.9	12.2	42.6	0	0.3	0
**GBS-04**	96.3	3.9	85.3	7.1	3.7	0	0	0	0	0
**GBS-05**	99.3	34.9	56.2	8.2	0.7	0	0	0	0	0
**GBS-06**	98.9	53.3	42.3	3.3	1.1	0	0	0	0	0
**GBS-07**	80.9	59.7	17.3	3.9	16.2	0	2.9	0	0	0
**GBS-08**	65.3	39.0	23.1	3.2	19.9	0	14.7	0	0	0
**GBS-09**	73.9	47.2	21.3	5.4	24.9	0	0.1	0	0	1.1
**GBS-10**	73.0	46.3	23.9	2.8	23.4	0	3.7	0	0	0
**GBS-11**	68.3	42.0	23.5	2.8	31.7	0	0	0	0	0
**GBS-12**	90.6	42.8	45.5	2.3	9.5	0	0	0	0	0
**GBS-13**	78.3	47.0	30.7	0.6	21.6	0	0.1	0	0	0
**GBS-14**	88.4	56.6	29.1	2.7	11.5	0	0	0	0	0
**GBS-15**	78.9	38.3	38.3	2.3	21.1	0	0	0	0	0
**GBS-16**	86.2	30.3	54.2	1.7	13.6	0	0	0	0	0
**DJB-01**	55.4	19.0	33.7	2.7	44.5	0	0	0	0	0
**DJB-02**	19.0	7.8	8.0	3.2	81.0	0	0	0	0	0
**DJB-03**	27.8	7.3	18.8	1.7	72.2	0	0	0	0	0
**DJB-04**	43.5	27.4	13.2	2.9	56.4	0	0	0	0	0
**DJB-05**	68.3	45.7	19.8	2.8	31.7	0	0	0	0	0
**DJB-06**	61.3	27.2	31.7	2.4	38.7	0	0	0	0	0
**DJB-07**	79.0	44.5	30.6	3.9	21.0	0	0	0	0	0
**DJB-08**	61.5	36.1	23.1	2.3	38.5	0	0	0	0	0
**DJB-09**	71.0	41.4	26.7	2.9	28.9	0	0	0	0	0
**DJB-10**	62.7	37.1	23.0	2.6	37.4	0	0	0	0	0
**DJB-11**	88.5	42.0	42.9	3.6	11.5	0	0	0	0	0
**DJB-12**	86.8	48.3	36.1	2.4	13.2	0	0	0	0	0
**DJB-13**	89.7	50.4	36.0	3.3	10.2	0	0	0	0	0
**DJB-14**	79.2	39.4	36.7	3.1	20.8	0	0	0	0	0
**DJB-15**	96.5	55.4	36.2	4.9	3.5	0	0	0	0	0

Along the Djerba transect, only four minerals were identified: calcite, Mg-calcite, aragonite and quartz (Fig 2). Calcite is minor component in all samples and its content (1.7–4.9 *wt*.%) does not show any important fluctuation. Mg-calcite is abundant in all samples with an average of 27.8 *wt*.%. Only sample in DJB-02 it reaches values of 8 *wt*.%. The maximum Mg-calcite content is reached in sample DJB-11 with 42.9 *wt*.%. Aragonite is the most abundant mineral with an average of 35.3 *wt*.%. Maximum aragonite content of 55.4 *wt*.% is reached at station DJB-15. Stations DJB-02 and DJB-03 show the lowest aragonite content, hoverer, its content increases from the coast to offshore. Quartz has an inverse trend and its content increases towards the shoreline (Fig 2). The maximum quartz content occurs at station DJB-02 with 81 *wt*.% and decreases to 3.5 *wt*.% at station DJB-15.

**Fig 6:**
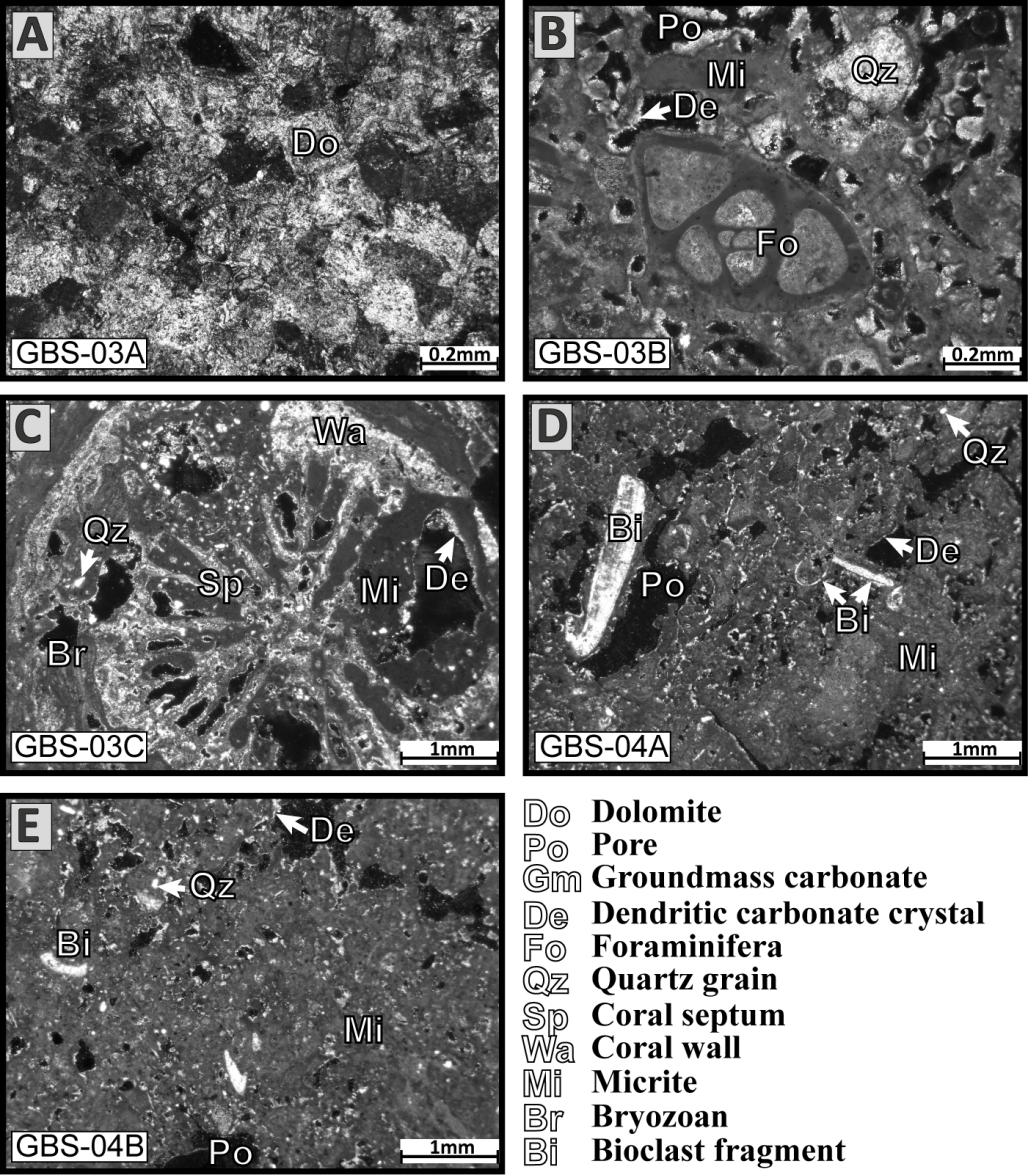
Thin section images of carbonate nodules. (A) Dolomite nodule from station GBS-03. (B) Nodule from station GBS-03 with bioclasts (foraminifera), quartz grains embedded in micrite. (C) Nodule from station GBS-03 with coral fragments and micrite. (D) Nodule from station GBS-04 with bioclasts, quartz grains into micrite. (E) Nodule from station GBS-04 with bioclasts, quartz grains embedded in micrite.

Nodule GBS-03A (Fig 6) is the only one composed of dolomite crystals. The shape of the crystals is relatively regular with rhombohedral shapes, however, some grains are rounded and a zonation may be visible. In a few cases, the centre is dissolved and only the rim of the crystal is remaining. This nodule does not contain quartz grains, bioclasts or pores. The carbonate nodule GBS-03B (Fig 6) has a high porosity and many rounded quartz grains and bioclasts (bivalves, foraminifera and bryozoan). Micrite is abundant and represents a large part of the nodule. Some pores have the shape of a bivalve fragment and indicate a total dissolution of bioclasts inside the nodule, in some cases the bioclast is partially dissolved. The internal edging of the pores is filled with very fine carbonate needles perpendicular to the porosity border. The nodule GBS-03C (Fig 6) is a coral fragment filled with micrite. The polished thin section is a cross section of the coral fragment showing wall, columella, septum and the external part covered by an encrusting bryozoan colony. The coral skeleton is micritic and pores are filled with micrite, rounded quartz grains, perpendicular carbonate needles also cover internal margins. Nodules from station GBS-04 are similar (Fig 6). Rounded quartz grains and bioclast are abundant and a large part of the nodules is micrite. The nodules contain large pores where perpendicular carbonate needles cover the internal margins and small pores are generally entirely filled by carbonate needles.

### Organic matter and phosphorus in surface sediment

**Fig 7:**
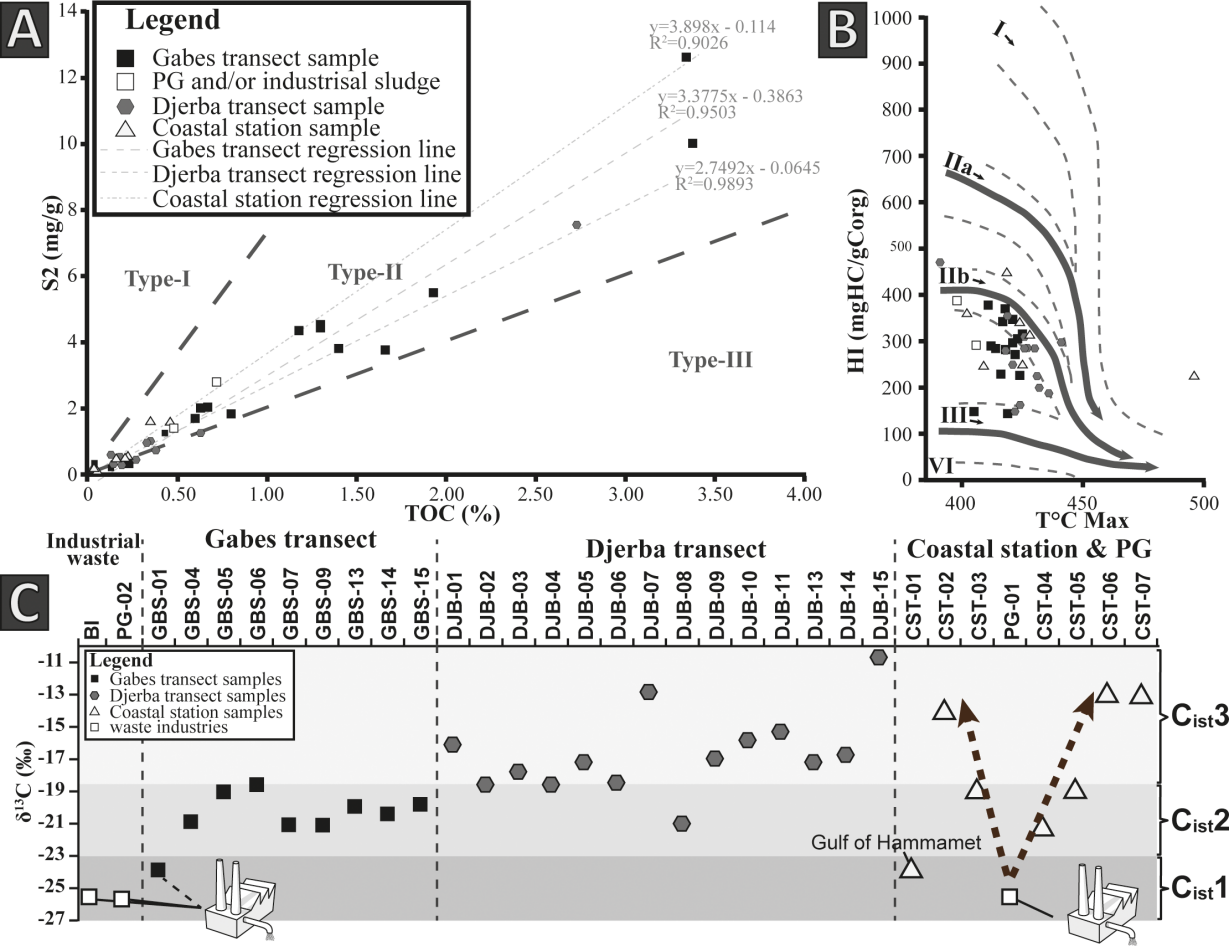
Origin of organic matter from surface sediments in the Gulf of Gabes. (A) S2 versus TOC diagram. (B) HI versus T max diagram. (C) The three clusters based on OM δ^13^C value.

Rock-Eval and C, H, N data are presented in Table 4. Along the Gabes transect, high TOC content is measured in samples GBS-02 and GBS-09 with values of 3.34 *wt*.% and 3.38 *wt*.%, respectively. Except for station GBS-02, TOC does not exceed 0.80 *wt*.% along the first seven kilometres of the Gabes transect. Along the rest of transect, TOC increases with values exceeding 1 *wt*.% (Fig 3). The HI and OI values vary from 143 to 378 mg HC/g TOC and 128 to 1083 mg CO_2_/g TOC, respectively. The average of TN content for the Gabes transect is 0.12 *wt*.% with a maximum of 0.33 *wt*.% at station GBS-09 and a minimum of 0.02 *wt*.% at station GBS-02. The total hydrogen content in samples from the Gabes transect is clearly higher than in the samples from the Djerba transect with an average of 0.47 *wt*.% and with a maximum of 0.99 *wt*.% at station GBS-09 and a minimum of 0.15 *wt*.% at station GBS-04. Highest matrix effect is reached in the Gabes transect samples with 0.3863 g of OM where absorbed per gram of sediment (Fig 7). Station GBS-05 has the lowest atomic C/N ratio of the whole Gabes transect. Atomic C/N ratio is especially high on the first two stations (GBS-01 and GBS-02) with molar ratio up to 24.7. The rest of samples from the Gabes transect (from GBS-05 to GBS-16) have molar C/N ratio slightly fluctuating from 16.3 (GBS-08) to 7.0 (GBS-15). Along the Djerba transect, only sample DJB-01 has a very high TOC content with 2.73% (Fig 3). In the remaining samples TOC does not exceed 0.63% (DJB-10) whereas, the average content is about 0.39 *wt*.%. Very low TOC contents are measured at stations DJB-02, DJB-03 and DJB-04 with values of 0.08 *wt*.%, 0.13 *wt*.% and 0.11 *wt*.%, respectively. The HI and OI values vary from 148 to 354 mg HC/g TOC and 273 to 2369 mg CO_2_/g TOC. Total nitrogen content is very low for all samples except for DJB-01 where the maximum is reached with 0.21 *wt*.%. Indeed the TN is around 0.02 *wt*.% for the majority of sample with an average of 0.04 *wt*.%. The same trend is observed for the total hydrogen content, where the maximum value is reached at sample DJB-01 with 0.53% while the average of the samples from the Djerba transect is around 0.21 *wt*.%. Matrix effect is relatively low for the sample from Djerba transect, with 0.0645 g of OM adsorbed per gram of sediment (Fig 7). Molar C/N ratios fluctuate significantly from 3.4 (DJB-06) to 16.3 (DJB-10), however most of the samples have molar ration close to 10 (DJB-02 to DJB-05; DJB-07; DJB-09; DJB-12; DJB-15). Total organic carbon in coastal samples is significantly lower than the samples from the Gabes transect but similar to the Djerba transect. It fluctuates from 0.05 to 0.35 *wt*.% and the maximum is reached at station CST-06 with 0.46 *wt*.%. The HI and OI vary from 224 to 359 mg HC/g TOC and 117 to 438 mg CO_2_/g TOC. Very low TN content not exceeding 0.06 *wt*.% characterizes coastal samples. In some stations, TN is below the detection limit (CST-01, CST-03, and CST-05). The total hydrogen content for coastal station samples varies from 0.01 to 0.20 *wt*.%. Matrix effect is the lower for the coastal stations with 0.114 g of OM adsorbed per gram of sediment (Fig 7). Industrial waste TOC values fluctuate from 0.04% (PG-02) to 0.72% (BI). The HI and OI values fluctuate significantly between the samples, from 292 mg HC/g TOC (PG-01) to 912 mg HC/g TOC (BI) and from 23 mg CO_2_/g TOC (PG-01) to 436 CO_2_/g TOC (PG-02). Total nitrogen content is low or equal to zero; however, waste industries are characterized by high total hydrogen content (up to 2.33%). Molar C/N ratio cannot be calculated for PG-01 and PG-02 because TN is below detection limit but the sample BI has the highest ratio (30.0) of the whole sample set.

**Table 4:** Geochemical data of OM and elemental composition of sediment.

Sample	Nitrogen	Carbon	Hydrogen	*δ*^13^C	MINC	TOC	HI	OI	T_max_	S1	S2	S3	C/N
**GBS-01**	0.02	0.43	0.32	-23.94	0.21	0.43	290	161	412	0.01	1.25	0.69	20.9
**GBS-02**	0.16	3.34	0.78	NA	6.38	3.34	378	128	411	0.07	12.62	4.26	24.7
**GBS-04**	0.03	0.13	0.15	-20.88	10.69	0.13	148	1083	405	0.00	0.20	1.43	5.8
**GBS-05**	0.03	0.23	0.19	-19.04	10.85	0.23	143	835	419	0.01	0.33	1.94	10.3
**GBS-06**	0.07	0.80	0.30	-18.58	10.32	0.80	229	333	416	0.01	1.82	2.65	13.1
**GBS-07**	0.06	0.60	0.29	-21.09	9.01	0.60	281	496	418	0.01	1.69	2.97	12.3
**GBS-08**	0.14	1.93	0.56	NA	7.54	1.93	284	233	414	0.02	5.49	4.50	16.2
**GBS-09**	0.33	3.38	0.99	-21.11	5.70	3.38	296	247	421	0.03	10.01	8.35	11.9
**GBS-10**	0.14	1.40	0.52	NA	7.56	1.40	271	359	422	0.02	3.80	5.02	12.1
**GBS-11**	0.17	1.30	0.48	NA	7.40	1.30	342	345	417	0.02	4.45	4.50	9.2
**GBS-12**	0.10	0.67	0.55	NA	8.99	0.67	305	566	423	0.01	2.03	3.76	8.0
**GBS-13**	0.16	1.66	0.51	-19.91	7.16	1.66	226	273	424	0.02	3.76	4.54	12.3
**GBS-14**	0.09	0.64	0.40	-20.44	9.12	0.63	315	572	425	0.01	2.00	3.63	8.1
**GBS-15**	0.20	1.18	0.49	-19.85	8.07	1.18	370	369	418	0.01	4.35	4.34	7.0
**GBS-16**	0.19	1.30	0.60	NA	7.15	1.30	347	393	421	0.02	4.51	5.11	8.1
**DJB-01**	0.21	2.73	0.53	-16.13	7.95	2.73	276	273	303	0.02	7.55	7.45	15.5
**DJB-02**	0.01	0.08	0.08	-18.59	3.58	0.08	279	1227	418	0.01	0.24	1.03	9.3
**DJB-03**	0.01	0.13	0.09	-17.78	4.94	0.13	470	849	391	0.01	0.59	1.06	10.8
**DJB-04**	0.01	0.11	0.11	-18.60	6.17	0.11	285	1303	427	0.01	0.32	1.48	10.7
**DJB-05**	0.03	0.18	0.16	-17.23	8.89	0.18	297	986	441	0.01	0.52	1.73	8.4
**DJB-06**	0.02	0.05	0.19	-18.46	0.28	0.05	354	2369	419	0.00	0.18	1.18	3.4
**DJB-07**	0.02	0.19	0.14	-12.82	8.41	0.19	148	991	422	0.01	0.27	1.84	11.1
**DJB-08**	0.02	0.15	0.17	-21.01	8.45	0.14	224	1475	431	0.01	0.32	2.14	6.8
**DJB-09**	0.04	0.38	0.20	-16.99	9.77	0.38	187	494	436	0.01	0.72	1.89	10.8
**DJB-10**	0.05	0.63	0.32	-15.84	8.55	0.63	199	687	432	0.02	1.25	4.30	16.3
**DJB-11**	0.05	0.35	0.27	-15.32	11.03	0.35	284	666	426	0.02	1.00	2.35	7.7
**DJB-12**	0.05	0.34	0.24	NA	11.24	0.33	284	638	430	0.01	0.95	2.14	8.2
**DJB-13**	0.04	0.21	0.23	-17.19	10.91	0.21	249	1093	421	0.01	0.52	2.28	6.1
**DJB-14**	0.03	0.13	0.18	-16.76	10.87	0.13	309	1082	426	0.00	0.41	1.44	4.7
**DJB-15**	0.03	0.27	0.18	-10.68	10.70	0.27	162	579	424	0.01	0.44	1.58	9.3
**CST-01**	0.00	0.05	0.01	-23.94	0.36	0.05	225	118	496	0.00	0.12	0.06	NA
**CST-02**	0.03	0.21	0.10	-14.12	1.78	0.21	246	321	409	0.01	0.51	0.67	9.1
**CST-03**	0.02	0.16	0.08	-19.02	4.66	0.16	313	439	428	0.01	0.49	0.69	11.7
**CST-04**	0.00	0.04	0.08	-21.25	1.93	0.04	360	396	402	0.00	0.15	0.16	NA
**CST-05**	0.00	0.22	0.15	-18.96	3.04	0.22	249	359	425	0.01	0.55	0.80	NA
**CST-06**	0.06	0.46	0.20	-13.10	6.24	0.46	341	366	424	0.01	1.58	1.69	8.8
**CST-07**	0.04	0.35	0.16	-13.15	6.68	0.35	461	376	420	0.01	1.61	1.31	9.3
**PG-01**	0.00	0.48	2.14	-25.53	0.06	0.48	292	24	406	0.02	1.40	0.11	NA
**PG-02**	0.00	0.06	2.33	-25.69	0.03	0.04	912	436	604	0.00	0.33	0.16	NA
**BI**	0.02	0.85	2.24	-25.58	0.12	0.72	387	41	398	0.04	2.79	0.29	30.0

Nitrogen, total carbon content and hydrogen contents (*wt*.%) δ^13^C of OM (‰), mineral carbon (MINC in %), Total Organic Carbon (TOC in %), Hydrogen Index (HI in mg HC/g TOC), Oxygen Index (OI in mg CO_2_/g TOC), Temperature maximum (T_max_ in°C), S1, S2 and S2b S3 peaks (mg HC/g) and molar C/N ratio.

Stable carbon isotopes data of OM (*δ*^13^C_OM_) are presented in Table 4, they vary from –25.6 to –10.7 ‰ (Fig 7). The average *δ* ^13^C_OM_ values of the Gabes transect samples is –20.2 ‰ with minimum values of –23.9 ‰ and maximum values of –18.6‰. Values from samples of the Djerba transect have higher *δ*^13^C_OM_ values ranging between –21.0 and –10.7 ‰. Lowest values are documented from the waste discharge site samples with values close to –25.6‰. In coastal stations, significant variations in values of *δ*^13^C are observed. Station CST-01, located in the Gulf of Hammamet has a *δ*^13^C_OM_ of -–23.9 ‰. Within the Gulf of Gabes, *δ*^13^C values decrease significantly close to the discharge area When at Skhira (CST-02) or at Djerba Island the *δ*^13^C_OM_ values are relatively high.

**Fig 8:**
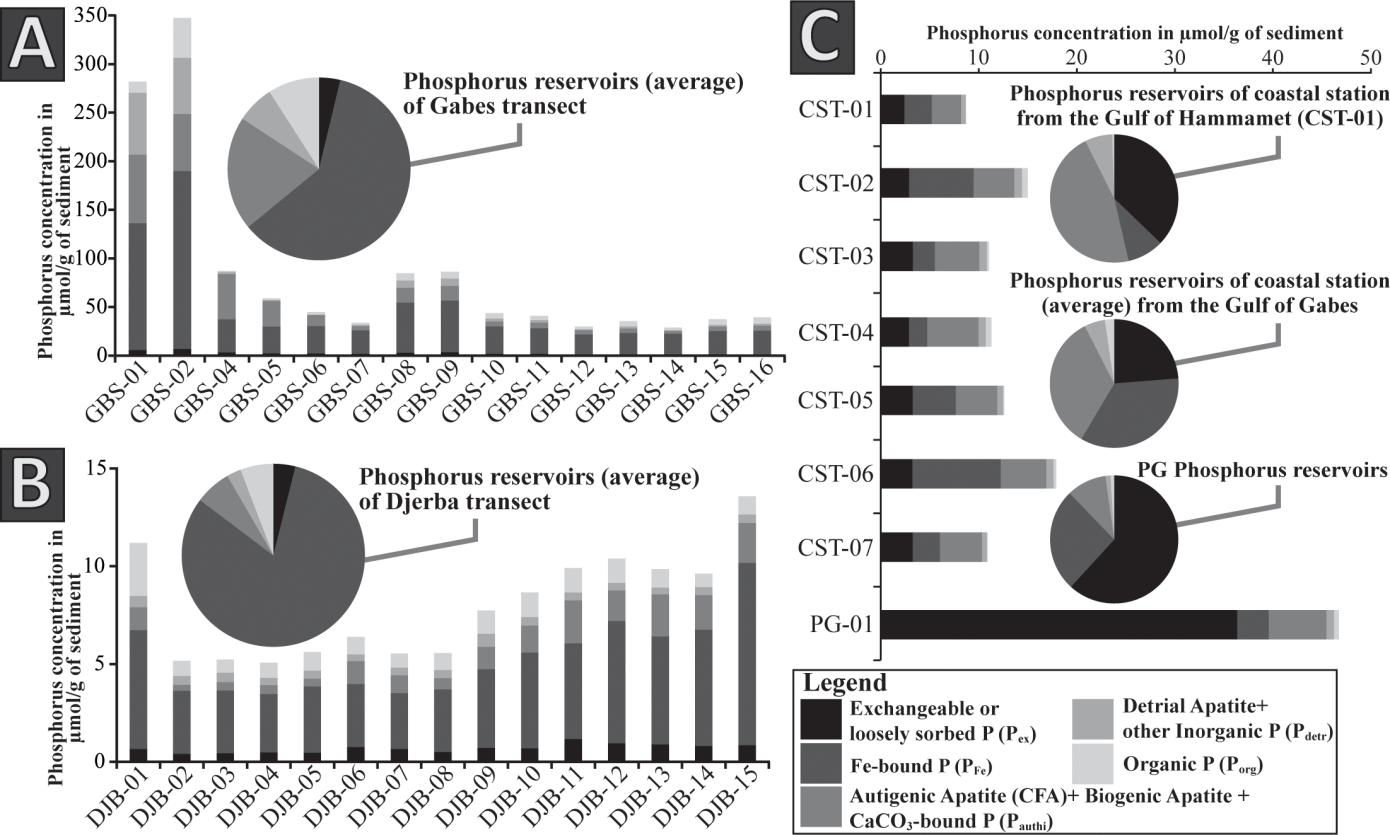
Phosphorus content and proportion in surface sediments from the Gulf of Gabes. (A) Phosphorus concentrations along the Gabes transect with the average phosphorus reservoirs. (B) Phosphorus concentrations along the Djerba transect with the average phosphorus reservoirs. (C) Phosphorus concentrations at coastal stations and phosphogypsum and phosphorus reservoirs of coastal stations from the Gulf of Hammamet, Gulf of Gabes and from phosphogypsum.

Phosphorus concentrations, proportions of the different phosphorus reservoir and molar ratios are in the Table 5. The P_total_ concentrations fluctuate considerably from 6.51 μmol P/g at the coastal station CST-01 to 347.64 μmol P/g at the station GBS-02. Concentrations are significantly higher along the Gabes transect (Figs 3 and 8), where the minimum value is reached at GBS-14 with 28.67 μmol P/g. Coastal stations and Djerba transect samples have similar concentrations with values fluctuating from 6.51 μmol P/g to 20.11 μmol P/g. Only PG-01 has concentrations reaching 58.85 μmol P/g. The P_ex_ concentrations are exceptionally high for waste industries with values up to 36.36 μmol P/g (Fig 8). All other samples have relatively low values from 0.41 μmol P/g for DJB-01 to 6.83 μmol P/g for GBS-02. Very high P_Fe_ concentrations are reached at station GBS-01 and GBS-02 (Fig 8) with respectively 130.7 μmol P/g and 183.23 μmol P/g. Except for these two stations, P_Fe_ concentration along the Gabes transect fluctuates from 20.5 μmol P/g (GBS-12) to 53.15 μmol P/g (GBS-09). Lower P_Fe_ concentrations were measured along the Djerba transect and at coastal stations with values between 2.86 μmol P/g (DJB-07) and 9.31 μmol P/g (CST-01). High P_authi_ concentrations were measured at station GBS-01 (Fig 8) with 70.61 μmol P/g. For the remaining samples of the Gabes transect, P_authi_ concentrations are high but range between 4.6 and 58.59 μmol P/g. P_authi_ concentrations on coastal stations are higher along the Djerba transect. The maximum P_authi_ concentration for the Djerba transect is reached at station DJB-13 with 2.15 μmol P/g whereas it reaches 5.22 μmol P/g at the coastal station CST-04. The P_detr_ concentrations are extremely high at stations GBS-01 and GBS-02 with respectively 63.56 μmol P/g and 57.95 μmol P/g. The remaining samples have relatively low concentrations with 0.35 μmol P/g (DJB-06) to 7.65 μmol P/g (GBS-09) (Fig 3). The P_org_ concentrations are very high at stations GBS-01 and GBS-02 with 11.47 μmol P/g and 41.05 μmol P/g, respectively. At all other stations, the P_org_ concentration does not exceed 7.69 μmol P/g. Highest P_ex_ proportion is in sample PG-01 at the waste discharge site (Fig 8) with 61.8 *wt*.%. However, P_ex_ proportions of coastal stations are significantly higher (between 21.8 and 37.2 *wt*.%) compared to the Djerba and Gabes transect samples, where they do not exceed 5.8 *wt*.%. Samples from the Djerba transect have the highest P_Fe_ proportion (Fig 8), between 74.8 and 86.3 *wt*.%. Gabes transect samples have relatively high proportion of P_Fe_ (between 46 and 68.7 *wt*.%) but the coastal station and samples from industrial waste sites have the lowest P_Fe_ proportion, especially CST-01 with only 9.2 *wt*.%. The P_authi_ proportions are especially high for coastal stations (between 31.3 and 46.2 *wt*.%) (Fig 8). Samples GBS-01 to -02, and GBS-04 to GBS-06 have significant high proportion of P_auti_ (>16.9 *wt*.%). Lowest P_authi_ proportions are reached for the Djerba transect and waste industries values do not exceed 10 *wt*.% (Fig 8). P_detr_ proportion is relatively low for the whole samples (between 1.3 and 8.8 *wt*.%) except for GBS-01 and GBS-02 with respectively 22.5 and 16.7 *wt*.% (Fig 8). Highest P_org_ proportion is reached for sample GBS-16 with 16.2 *wt*.%. Except for some samples (GBS-02; GBS-10; GBS-11; GBS-14 and GBS-16; DJB-01) with values higher than 10 *wt*.%, P_org_ proportion is relatively low (Fig 8). Molar C/P ratio is relatively high (up to 3930.9) for coastal station samples except for CST-04 with a ratio of 57.2. Molar C/P ratio for Gabes transect, Djerba transect and waste industries samples, significantly fluctuate. Lowest C/P ratio with respectively 31.2 and 67.8 occur in samples GBS-01 and GBS-02, while highest ratio of 837.9 and 786.2 were measured for samples DJB-01 and PG-01. However, Fe/P_Fe_ ratio is very low for the whole samples set, except for the coastal stations CST-01, CST-02 and CST-06, it does not exceed 0.8.

**Table 5:** Phosphorus reservoirs distribution in surface sediments.

			Phosphorus reservoir concentration [μmol P/g]							Phosphorus reservoir proportion [%]					Molar ratios		
Sample number		Distance from the coast (km)	P_ex_	P_Fe_	Fe	P_authi_	P_detr_	P_org_	P_total_	P_ex_	P_Fe_	P_authi_	P_detr_	P_org_	C/P	P_Fe_/Fe	Fe/P_Fe_
**GBS-01**		0.4	5.60	130.70	12.15	70.61	63.56	11.47	281.94	2.0	46.4	25.0	22.5	4.1	31.2	10.8	0.1
**GBS-02**		1.5	6.83	183.23	14.86	58.59	57.95	41.05	347.64	2.0	52.7	16.9	16.7	11.8	67.8	12.3	0.1
**GBS-04**		3.5	3.24	34.02	10.39	46.45	2.24	1.12	87.07	3.7	39.1	53.3	2.6	1.3	97.0	3.3	0.3
**GBS-05**		4.4	2.43	27.54	13.28	26.05	1.25	1.64	58.91	4.1	46.7	44.2	2.1	2.8	116.6	2.1	0.5
**GBS-06**		5.4	2.15	28.28	9.05	11.17	0.79	2.29	44.68	4.8	63.3	25.0	1.8	5.1	291.6	3.1	0.3
**GBS-07**		6.4	1.90	24.11	7.69	4.45	0.98	2.17	33.61	5.7	71.7	13.2	2.9	6.5	230.3	3.1	0.3
**GBS-08**		7.6	3.02	51.58	12.11	15.07	7.42	7.69	84.78	3.6	60.8	17.8	8.7	9.1	209.1	4.3	0.2
**GBS-09**		8.7	3.48	53.15	19.82	15.04	7.65	7.10	86.40	4.0	61.5	17.4	8.8	8.2	396.9	2.7	0.4
**GBS-10**		9.8	1.85	28.23	11.73	4.99	3.05	5.65	43.77	4.2	64.5	11.4	7.0	12.9	206.4	2.4	0.4
**GBS-11**		10.7	1.72	26.38	11.63	5.80	2.63	4.30	40.83	4.2	64.6	14.2	6.4	10.5	251.8	2.3	0.4
**GBS-12**		11.8	1.21	20.50	6.53	4.52	0.84	2.77	29.84	4.1	68.7	15.1	2.8	9.3	201.8	3.1	0.3
**GBS-13**		12.7	1.34	21.75	8.42	4.87	2.20	5.13	35.29	3.8	61.6	13.8	6.2	14.5	269.9	2.6	0.4
**GBS-14**		13.6	1.06	21.59	7.81	2.89	0.65	2.49	28.67	3.7	75.3	10.1	2.3	8.7	211.2	2.8	0.4
**GBS-15**		14.7	1.16	24.15	11.92	4.60	1.59	5.68	37.17	3.1	65.0	12.4	4.3	15.3	173.2	2.0	0.5
**GBS-16**		17.3	1.32	24.45	10.90	5.19	1.89	6.37	39.20	3.4	62.4	13.2	4.8	16.2	170.2	2.2	0.4
**Average (Gabes transect)**		-	2.55	46.64	11.22	18.69	10.31	7.13	85.32	3.8	60.3	20.2	6.7	9.1	195.0	3.9	0.3
**DJB-01**		0.6	0.66	18.38	6.07	1.17	0.58	2.72	23.50	2.8	78.2	5.0	2.5	11.6	837.9	3.0	0.3
**DJB-02**		1.7	0.41	12.18	3.22	0.30	0.46	0.77	14.12	2.9	86.3	2.1	3.3	5.4	87.1	3.8	0.3
**DJB-03**		2.5	0.44	11.94	3.21	0.42	0.49	0.67	13.96	3.1	85.5	3.0	3.5	4.8	161.0	3.7	0.3
**DJB-04**		3.5	0.48	12.29	2.99	0.46	0.37	0.77	14.36	3.3	85.6	3.2	2.6	5.3	119.8	4.1	0.2
**DJB-05**		4.4	0.46	13.89	3.39	0.39	0.42	0.95	16.11	2.9	86.2	2.4	2.6	5.9	158.2	4.1	0.2
**DJB-06**		5.1	0.75	11.95	3.22	1.17	0.35	0.90	15.12	5.0	79.0	7.7	2.3	6.0	46.2	3.7	0.3
**DJB-07**		6.0	0.65	13.02	2.86	0.91	0.40	0.72	15.69	4.2	83.0	5.8	2.5	4.6	220.2	4.6	0.2
**DJB-08**		7.1	0.51	13.66	3.19	0.58	0.42	0.86	16.03	3.2	85.3	3.6	2.6	5.3	136.3	4.3	0.2
**DJB-09**		8.2	0.71	16.15	4.02	1.15	0.67	1.18	19.86	3.6	81.3	5.8	3.4	5.9	268.8	4.0	0.2
**DJB-10**		9.1	0.69	14.81	4.89	1.38	0.44	1.26	18.58	3.7	79.7	7.4	2.4	6.8	417.0	3.0	0.3
**DJB-11**		10.4	1.17	14.96	4.88	2.21	0.39	1.26	19.99	5.8	74.8	11.1	2.0	6.3	231.7	3.1	0.3
**DJB-12**		11.1	0.95	15.91	6.25	1.56	0.39	1.24	20.05	4.7	79.4	7.8	2.0	6.2	222.5	2.5	0.4
**DJB-13**		12.4	0.89	15.22	5.52	2.15	0.35	0.94	19.54	4.5	77.9	11.0	1.8	4.8	185.4	2.8	0.4
**DJB-14**		13.1	0.82	14.57	5.94	1.76	0.42	0.70	18.26	4.5	79.8	9.6	2.3	3.8	155.7	2.5	0.4
**DJB-15**		13.8	0.86	15.86	9.31	2.04	0.44	0.92	20.11	4.3	78.8	10.1	2.2	4.6	244.3	1.7	0.6
**Average (Djerba transect)**		-	0.70	14.32	4.60	1.18	0.44	1.06	17.69	3.9	81.4	6.4	2.5	5.8	232.8	3.4	0.3
**CST-01** **Gulf of Hammamet**		0.0	2.42	0.60	2.80	3.01	0.45	0.03	6.51	37.2	9.2	46.2	6.9	0.5	1310.3	0.2	4.7
**CST-02**	**Gulf of Gabes**	0.0	2.91	4.08	6.58	4.14	0.79	0.58	12.50	23.3	32.6	33.1	6.3	4.7	300.2	0.6	1.6
**CST-03**		0.0	3.31	4.73	2.23	4.52	0.78	0.21	13.55	24.4	34.9	33.4	5.8	1.6	628.9	2.1	0.5
**CST-04**		0.0	2.90	3.89	1.87	5.22	0.73	0.58	13.32	21.8	29.2	39.2	5.5	4.4	57.2	2.1	0.5
**CST-05**		0.0	3.28	5.34	4.40	4.23	0.58	0.11	13.54	24.2	39.5	31.3	4.3	0.8	1729.6	1.2	0.8
**CST-06**		0.0	3.26	5.18	9.00	4.64	0.74	0.28	14.10	23.1	36.7	32.9	5.2	2.0	1390.9	0.6	1.7
**CST-07**		0.0	3.27	4.57	2.77	4.29	0.51	0.07	12.71	25.7	35.9	33.7	4.0	0.6	3930.8	1.7	0.6
**Average (Gulf of Gabes)**		-	3.16	4.63	4.48	4.51	0.69	0.31	13.29	23.8	34.8	33.9	5.2	2.4	1339.6	1.4	1.0
**PG-01**		0.0	36.36	15.33	3.21	5.90	0.75	0.51	58.85	61.8	26.0	10.0	1.3	0.9	786.2	4.8	0.2

Absolute (in μmol P/g) and relative content (in %) of: Exchangeable or loosely absorbed P (P_ex_); Fe-Bound P (P_Fe_); Authigenic P (P_authi_); Detrital P (P_detr_) and Organic P (P_org_). Are also shown Fe concentration (μmol P/g) and molar C/P, P_Fe_/Fe and Fe/P_Fe_ ratios.

## Discussion

### Signs of eutrophication in the Gulf of Gabes

Eutrophication is defined by Nixon [49] as “an increase in the rate of supply of OM to an ecosystem”. Eutrophication results from a nutrient enrichment and induces an intensification of all biological activities causing changes in the ecosystems [50]. Nitrogen and phosphorus profoundly influence the marine environment and increase the algae productivity [51].

Smith [52] lists eutrophication effects and many of them have been observed along the Gabes transect:

▪ A decrease in water column transparency is one of the most visible eutrophication effects observed along the Gabes transect.
▪ Depletion of oxygen in the water was recorded along the Gabes transect (Table 2, Fig 2).
▪ Changes in composition of marine vascular plants. At the beginning of the 20^th^ century, the sea floor in Gulf of Gabes was almost entirely colonized by *P*. *oceanica* [40]. It was estimated that 90% of this cover disappeared around 1960 [39]. No *P*. *oceanica* was documented along the Gabes transect contrary to the Djerba transect especially in its proximal part (Fig 5). The decline of *P*. *oceanica* in the Gulf of Gabes is probably related to the phosphate industries pollution [39, 40, 53].
▪ The decline of the coral *Cladocora caspidosa* in the inner part of the Gulf of Gabes during the last 30 years [28]. Presently living *C*. *caespitosa* is found only SE of Kerkennah Islands and NE of Djerba [54].

**Fig 9:**
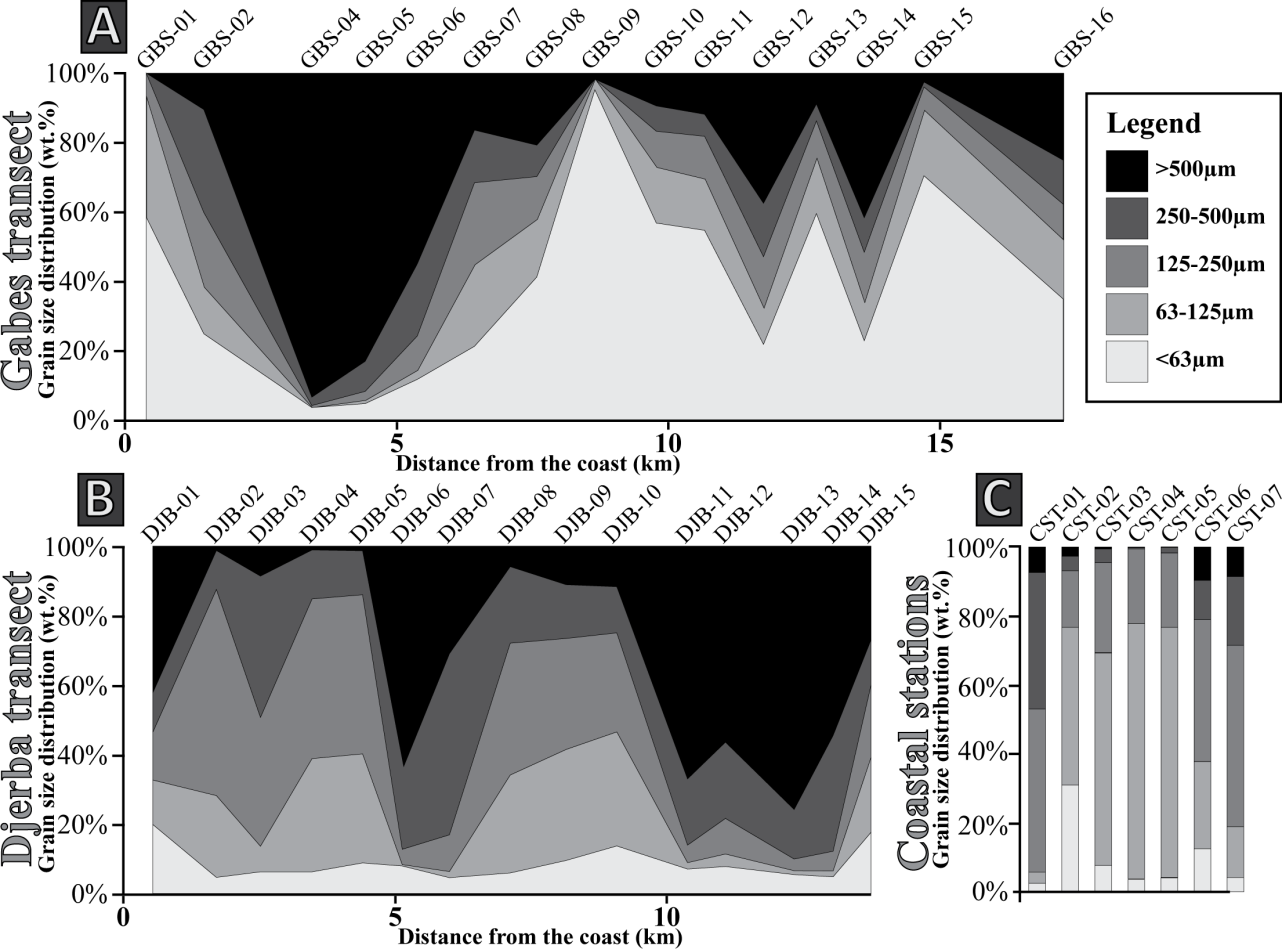
Grain size distribution. (A) Gabes transect. (B) Djerba transect. (C) Coastal stations.

Another eutrophication effect observed and not mentioned by Smith [52] is the siltation of the seafloor. It has been described for the first time in 1976, only a few years after the starting of the production of phosphoric acid at Gabes [55]. Since the seventies, the seafloor has considerably changed: at the beginning of the eighties, the water within the first three kilometres of the waste discharge area was acidic without any algae and/or marine plants. *Caulerpa prolifera* replaced *P*. *oceanica* at 3 km from the coast to offshore [19]. In 1990, Zaouali [40] noticed severe degradation. Living algae and/or aquatic plants were totally absent at the seafloor but remains of *P*. *oceanica* rhizome were found in surface sediments. In 2014, environmental conditions deteriorated and the siltation progressed. A large part of the sediments along Gabes transect became finer (Fig 9) and the fragments of dead rhizomes of *P*. *oceanica* disappeared (Figs 4 and 5). In addition, the abundance of the bivalve *Corbula gibba* in the distal part of the Gabes transectis indicative of siltation and points toward a polluted environment. In fact, increased abundances of *C*. *gibba* have been reported from environments with high OM accumulation, low oxygen and high turbidity [56].

### Evidences of heavy contamination adjacent to the industrial waste discharge

Heavy metals contamination by phosphate industries in the Gulf of Gabes is demonstrated since several decades (e.g. [57, 58]). Recent studies document a strong zinc contamination [26, 27, 53] here confirmed by the presence of sphalerite mineral in surface sediments (Table 3, Fig 2). Our XRD data set confirm and give supplementary evidence of heavy metal contamination. A large amount of gypsum, having the same mineralogical composition of PG [15, 16] (Table 3, Fig 2) is observed in the proximal part of the Gabes transect. Several studies have shown the heavy metals content [12, 13, 17, 22] and the radioactive properties of PG [12, 16, 59]. In Gabes, PG represents an additional risk of large-scale contamination because is highly soluble in seawater [60] and can liberate different pollutant compounds into the environment, which are transported by marine currents.

Low seawater pH near the waste discharge (Table 2, Figs 2 and 3) is an additional sign of heavy pollution. Normal marine surface seawater pH is around 8.2 [61], below this value it can have a major impact on the environment, especially on calcifying organisms [62]. Our study shows a direct impact of the acid discharge on the sedimentary facies with formation of carbonate nodules (Figs 5 and 6). At high pH the dominant carbon species is CO_3_^2-^ inducing the formation of carbonate minerals [63, 64]. The increase in pH at the proximal section of the Gabes transect may be responsible for carbonate needles precipitation and formation of carbonate nodules, starting from a nucleation point consisting of bioclasts. Nodules are highly micritic (Fig 6). The micritization process is the alteration of carbonate grains by boring algae filling the borings/holes with micritic material [65, 66]. However, the micritization processes can be also the result of bacteria [67]. This hypothesis seems to be confirmed by the low atomic C/P ratio typical of microbial activity (Table 5) at the proximal part of the Gabes transect (e.g. [30, 68]). The dolomitic nodule (Fig 6) could be an additional evidence of microbial activity at the proximal section of Gabes transect. Dolomite formation can occur under different conditions including within microbial mats [69] as bacteria can participate to primary dolomite formation. Modern examples are in shallow and/or intertidals such as Lagoa Vermelha (Brazil) [70] or in coastal sabkhas, e.g., of Abu Dhabi [71]. However, the interpretation of the genesis of the nodules is not straightforward: the atomic C/P ratio may not necessarily reflect microbial activity because of the strong influence of industrial waste discharge along the proximal section of Gabes transect. In addition, the sugar-like crystalline structure of the dolomite nodule (Fig 6) may also imply a primary chemical precipitation or a secondary replacement [70]. Laminated structures resulting from microbial mats are generally present in modern dolomite [70, 71]. The dolomitic nodule (Fig 6) may be a fragment discharged by the phosphate industries via the canal discharging the industrial waste. Dolomites from Lower Cretaceous outcrops are found in Tunisia [72] around the Gafsa basin close to the mining site [73, 74].

### Organic matter: Origin and pollution indicator

As food for benthic organism, the contribution and the source of the marine OM may be a key factor for biodiversity (e.g. [75]). Organic matter can also indicate an eutrophication process in marine environment due to anthropogenic activities [49]. Variations of OM input into the environment may change many chemical, physical and biological processes and have a direct impact on the fauna [76].

High OM along the Gabes transect may be a sign of eutrophication. However, since sedimentary OM is strongly influenced by particle size [77] the preservation of OM at Gabes may be linked to fine sediments (Fig 9). The mineralogical composition of sediments plays also a role in OM preservation. High specific surface of clay minerals allow a better adsorption of OM [78]. We detected kaolinite along the Gabes transect (Table 3) and Ben Amor et al. [79] documented high clay mineral contents in the inner part of the Gulf. Higher matrix effect would confirm a higher proportion of clay mineral in Gabes than in the other stations. Clay minerals in the sediments are generally responsible for matrix effect [42, 44], however, other minerals like gypsum can induce a matrix effect even in small quantity (e.g., 10 *wt*.%) [80]. The presence of PG along the Gabes transect (Table 3, Fig 2) has probably induced matrix effects due to the similar mineralogy properties as gypsum [15, 16].

A large part of OM documented in the proximal part of the Gabes transect belongs to the industrial waste discharge. Indeed, dark industrial sludge rich in OM (Table 4) was observed around the waste discharge area (waste discharge canal, seafloor of GBS-02 and GBS-03 stations). This OM is directly related to phosphorite ore used for the phosphate treatment. Belayouni and Trichet [6] showed that the phosphorites from the Gafsa basin are relatively rich in OM [7]. During the pre-treatment, the phosphorite ore is enriched in phosphorus by several crushing and sieving steps [81]. During phosphoric acid production, all impurities (e.g., OM, heavy metals, non-dissolved minerals) are concentrated into industrial sludge and discharged into the waste canal and carried by the current to reach the seafloor at station GBS-02.

Hydrogen Index vs. Oxygen Index is generally used to determine the kerogen type and the origin of sedimentary OM, however, high OI represent a serious limitation in identifying OM origin. High OI values (Table 4) can be related to a very low TOC, which induce an adsorption of CO_2_ [82]. In addition, high carbonate contents in sediments (Table 3) increase significantly the OI due to the carbonate dissociation during the heating process of the Rock-Eval analysis [45]. Therefore, S_2_ vs. TOC and HI vs. T°_max_ diagrams are used to interpret and to determine the OM origin in the Gulf of Gabes. Both graphs (Fig 7) indicate type II kerogen (mixed marine and terrestrial origin) and reflect the complexity of the different OM source along coastal environment.

Terrestrial sedimentary OM contribution can be possibly due to the proximity of the Gabes oasis and/or be related to the numerous wadis present along the Gulf of Gabes. However, *P*. *oceanica* which is abundant in Djerba (Figs 4 and 5) share common characteristics with land plants, e.g., it possesses cuticles and contains a high amount of cellulose [82, 83] and therefore, it may give the same kerogen signature as terrestrial OM (Fig 7). Marine OM origin is mainly due to zoo- and phytoplankton, which may also contribute to the turbidity of seawater. The large amount of OM contained in the industry waste near the industrial complex of Gabes can bias the interpretation of OM origin. Several studies [6, 7, 84] indicate a marine origin of the OM from the Gafsa phosphorites.

The estimation of the different marine vs. terrestrial contributions in OM is not possible with Rock-Eval. Additional information is derived from stable carbon isotope composition of OM. These analyses indicate three clusters with different OM origin: C_ist_1, C_ist_2 and C_ist_3 (Fig 7).

Cluster C_ist_1 is characterized by depleted δ^13^C values close to -25‰ and it groups samples from the area of industrial discharge (sediments and industrial waste) (Fig 7). The coupling δ^13^C_OM_ and molar C/N ratio clearly shows the influence and the contribution of the waste discharge in the Gulf of Gabes, especially around the industrial waste discharge area (Table 4, Fig 7). In particular, the isotopic signature of OM from the industrial waste (around -25‰) corresponds to the isotopic composition of OM from the phosphorite from the Gafsa basin (-26.5‰ to -24‰) [11]. Depleted δ^13^C_OM_ in the past is common, for example, marine OM from Cretaceous rocks has typical values of -28‰ to -27‰, while modern marine sediments have values around -22‰ [85]. Other studies also indicate a worldwide depletion of δ^13^C from the Palaeocene to the Eocene [86, 87].

Depleted OM δ^13^C can be also caused by diagenesis. Freudenthal et al. [88] refer to two processes at the origin of changes in its isotopic composition. These include the preferential degradation of organic compound, which may cause a negative shift of δ^13^C and the fractionation of stable carbon isotopes by organisms (e.g., bacteria), which degrades OM in the sediment. The very high molar C/N ratio for the industrial waste (Table 4) suggests loss in nitrogen during diagenesis (e.g., [89, 90]). Total nitrogen is not obviously related to the OM but can be captured by the crystal lattice of minerals present in the sedimentary rock [91]. The mineralized nitrogen is dissolved during the phosphorite treatment and not retained in the industrial waste, inducing a high C/N ratio. However, mineralized nitrogen seems low in phosporite from Gafsa because Belayouni et al. [7] noticed a high molar C/N ratio (from 25.1 to 90.5) in the humic compound.

Cluster C_ist_2 is characterized by intermediate δ^13^C_OM_ values between -23‰ and -18.5‰ (Fig 7). This δ^13^C signature corresponds to marine phytoplankton OM [92, 93]. Generally, molar C/N ratio for plankton is between 7 and 9 [94, 95], thus it is lower than the C/N ratio measured in this cluster. Therefore, a probable OM contribution from the waste discharge in the distal area of Gabes transect and coastal stations cannot be excluded. The marine phytoplankton δ^13^C signature may reflect a phosphorus contamination in the Gulf of Gabes. Phosphorus contained in PG is release directly into seawater. As a nutrient, phosphorus is a limiting factor to the primary producers [96–98] and increases phytoplankton production. Indeed, 176 phytoplankton blooms were recorded between 1995 and 2005 and represent high frequency events [99].

Cluster C_ist_3: Enriched δ^13^C_OM_ values (>-18.5‰) are typical for C4 land plant [95, 100] and Rock-Eval data suggest an OM terrestrial source (Fig 7). However, the low C/N ratio in sediments does not correspond to terrestrial OM and higher plant (>12 for [94] and between 20 and 500 for [95]). The abundance of *P*. *oceanica* meadows along the Djerba transect (Figs 4 and 5) and along coastal station is a possible OM source. Stable carbon isotope composition of *P*. *oceanica* ranges from -19.7‰ to -10.8‰ [101–104], which corresponds to the values measured for this cluster (Table 4, Fig 7). However, molar C/N ratio of *P*. *oceanica* (27.4 in [102]) is considerably higher than the ratios of this cluster. Garcias-Bonet et al. [105] detected the presence of nitrogen-fixing symbiotic bacteria in *P*. *oceanica* roots, which could decrease the molar C/N ratio.

Stable carbon isotope composition of sedimentary OM matter reflects three impacted areas by phosphate industries with different pollution levels delimited by the three clusters C_ist_1, C_ist_2 and C_ist_3.

### Phosphorus in the Gulf of Gabes: A pollutant and its implication in the phosphorus cycle

The distribution of total phosphorus concentrations shows a similar area of pollution level delimited by δ^13^C clusters (Fig 3). However, increases in P_total_ concentrations from the coast to the open sea along the Djerba transect (Table 5, Fig 8) indicate a much larger-scale phosphorus contamination. Previous studies indicated heavy metals contamination from Gabes to the southwestern coast of its Gulf [26] and in the Boughrara lagoon [106]. Marine currents are responsible for the heavy metal dispersion [26, 106, 107] as well as for the phosphorus dispersion from Gabes to the offshore of Eastern Djerba coast.

The large amount of PG discharged into the seawater has negative impact on the phosphorus cycle. Indeed part of the phosphorus released by PG precipitates into autigenic phosphorus (P_authi_) along the Gabes transect. High concentration and proportion of P_ex_ in PG represent the residual phosphorus from the phosphorite treatment by sulphuric acid. Phosphorite is mostly composed by authigenic apatite (pellets and coprolites) and biogenic apatite (sharks and rays fossil teeth) [9, 11], represented by P_auth_, and it is dissolved during the industrial treatment. Even P_Fe_ from phosphorite is dissolved because P_Fe_ is easily reducible [29] and ferric oxides are soluble in sulphuric acid (e.g. [108]). Detrital apatite (P_detr_) seems relatively resistant to the industrial treatment because high concentration of P_detr_ is noticed around the industrial waste discharge (Table 5, Fig 8). An accumulation of detrital apatite released by PG occurs by gravitational segregation due to the higher specific gravity of apatite compared with other minerals (e.g. quartz, calcite, aragonite, gypsum) [109]. Residual phosphorus in solution from the industrial treatment is adsorbed by PG and released in seawater because of high PG solubility in seawater (around 4.1 g/l) [17] and high magnesium and chloride concentration in seawater. These two abundant elements allow bringing P_ex_ in solution by formation of MgPO_4_^-^ or by mass action displacement [29]. Even if high sedimentary P_authi_ concentration along the Gabes transect (Fig 8) could be linked to an accumulation effect from PG discharge, an in-situ authigenic phosphorus precipitation at the sediment-seawater interface cannot be excluded [110]. Authigenic apatite precipitation requires fluorine, calcium, magnesium, and sulphate from seawater and high phosphorus concentration in pore waters. Phosphorus is normally released in pore waters by microbial degradation of OM from the sediment. Pore waters saturated in Fe^2+^, PO_4_^3-^ and F^-^ provide the conditions suitable for authigenic apatite precipitation [34]. However, Tunisian PG contains phosphorus (1.69 *wt*.%), fluorine (0.55 *wt*.%) and iron (0.03 *wt*.%) [15] that can be released into the seawater through their solubility and allow further authigenic apatite precipitation.

Ferric oxyhydroxide minerals in sediments have the property to adsorb phosphorus, increase its concentration in the sediment and promote formation of P_authi_ [34] along the Gabes transect. Although, P_Fe_ concentration is significantly higher along the Gabesthan Djerba transects (Fig 8), the P_Fe_/Fe ratio is similar (Table 5) and may indicate similar ferric oxyhydroxide iron mineral phases between the two transects. Molar Fe/P ratios in this study are extremely low (Table 5) compared to other localities, such as the Labrador Sea (between 20 and 26 in [111]) or the Iberian margin (between 6 and 25 in [112]). Van der Zee et al. [112] suggest that low molar Fe/P ratio is the result of high phosphorus adsorption of poorly crystalline ferric oxyhydroxide mineral phases having a larger surface capacity and higher adsorption capacity [113]. In addition, high P_Fe_ concentration in the Gulf of Gabes compared to the Labrador Sea and the Iberian margin [111, 112] reflect a well-oxygenated environment, which increase significantly phosphorus adsorption on ferric oxyhydroxide minerals [112, 114, 115].

Phosphorus reservoirs proportion along the coastal stations might reflect the phosphorus reservoirs composition of the bedrock due to important coastal erosion along Tunisian coasts [116]. The large difference in phosphorus reservoir proportion between the Gulf of Hammamet and the Gulf of Gabes (Table 5, Fig 8) can be related to the age of the rock substratum. The southern part of the Gulf of Hammamet have mainly a Miocene-Pliocene substratum while the Gulf of Gabes and the Djerba Island the substratum is composed by Quaternary rocks [73, 74]. However, formation of modern authigenic phosphorus cannot be excluded due to the large contribution of P_ex_ and P_auth_ (Fig 8). Sheldon [117] have previously suggested formation of authigenic phosphorus at the water-sediment interface in coastal environments. Autigenic apatite precipitation on coastal environment could be related to the high concentration and high proportion of P_ex_ in sediments (Fig 8). Al-Enezi et al. [118] showed a positive correlation between the quartz and the phosphorus adsorption, however other factors such as pH and salinity may also influence phosphorus adsorption in sediments [118–120]. Authigenic apatite precipitation can be influenced by parameters such as temperature, pH and adsorbed Mg^2+^ ions [121]. Significant variations in seawater temperature and salinity occurring between winter and summer in the Gulf of Gabes [53, 122, 123] could induce autigenic precipitation.

## Conclusions

This study presents the impact of phosphate treatment waste discharge into the marine environment in Tunisia. Different impacted areas are identified mostly based on the δ^13^C signature of sedimentary OM and on the P_total_ concentration as follows:

1. The area close to the phosphate industry complex is severely impacted by the industrial waste discharge and the environmental condition can be considered as critical.
2. The Gabes off shore is also strongly impacted by the phosphate industries.
3. The first signs of phosphorous contamination by industrial waste discharge are observed in Djerba.

The high volume of industrial waste discharge has serious consequences on the marine environment in the inner part of the Gulf of Gabes: OM and detrital apatite accumulation, acidic seawater with formation of carbonate nodules and severe eutrophication of marine environment. The large PG discharge impacts also the phosphorus cycle with a high authigenic phosphorus precipitation especially in the Gulf of Gabes.

## References

[1] Ruttenberg KC. Reassessment of the oceanic residence time of phosphorus. Chemical Geology. 1993; 107: 405–409.

[2] Föllmi KB. The phosphorus cycle, phosphogenesis and marine phosphate-rich deposits. Earth-Science Reviews. 1996; 40: 55–124.

[3] Ruttenberg KC, Dyhrman ST. Dissolved organic phosphorus production during simulated phytoplankton blooms in a coastal upwelling system. Frontiers in Microbiology. 2012; 3: 1–12. doi: 10.3389/fmicb.2012.00001 22888326 10.3389/fmicb.2012.00274 PMC3412337

[4] Cisse L, Mrabet T. World Phosphate Production: Overview and Prospects. Phosphorus Research Bulletin. 2004; 15: 21–25.

[5] Notholt AJG, Sheldon RP, Davidson DF. Phosphate deposits of the world, volume 2 Phosphate rock resources. Cambridge University Press; 2005.

[6] Belayouni H, Trichet J. Hydrocarbons in phosphatized and non-phosphatized sediments from the phosphate basin of Gafsa. Organic Geochemistry. 1984; 6: 741–754.

[7] Belayouni H, Slansky M, Trichet J. A study of the organic matter in Tunisian phosphates series: Relevance to phosphorite genesis in the Gafsa Basin (Tunisia). Organic Geochemistry. 1990; 15(1): 47–72.

[8] Beji Sassi A, Sassi S. Cadmium associated with phosphate deposits in southern Tunisia. Journal of African Earth Sciences. 1999; 29 (3): 501–513.

[9] Ounis A, Kocsis L, Chaabani F, Pfeifer HR. Rare earth elements and stable isotope geochemistry (δ^13^C and δ^18^O) of phosphorite deposits in the Gafsa Basin, Tunisia. Palaeogeography, Palaeoclimatology, Palaeoecology. 2008; 268: 1–18.

[10] Kocsis L, Ounis A, Chaabani F, Neili MS. Paleoenvironmental conditions and strontium isotope stratigraphy in the Paleogene Gafsa Basin (Tunisia) deduced from geochemical analyses of phosphatic fossils. International Journal of Earth Sciences (GR Geologische Rundschau). 2013; 102: 1111–1129.

[11] Kocsis L, Ounis A, Baumgartner C, Pirkenseer C, Harding IC, Adatte T et al Paleocene–Eocene palaeoenvironmental conditions of the main phosphorite deposits (Chouabine Formation) in the Gafsa Basin, Tunisia. Journal of African Earth Sciences. 2014; 100: 586–597.

[12] Rutherford PM, Dudas MJ, Samek RA. Environmental impacts of phosphogypsum. The Science of the Total Environment. 1994; 149: 1–38.

[13] Zairi M, Rouis MJ. Impacts environnementaux du stockage du phosphogypse à Sfax (Tunisie). Bulletin des laboratoires des Ponts et Chaussées. 1999; 219: 29–40.

[14] Pérez-López R, Álvarez-Valero M, Nieto JM. Changes in mobility of toxic elements during the production of phosphoric acid in the fertilizer industry of Huelva (SW Spain) and environmental impact of phosphogypsum wastes. Journal of Hazardous Materials. 2007; 148: 745–750. doi: 10.1016/j.jhazmat.2007.06.068 17683858 10.1016/j.jhazmat.2007.06.068

[15] Ajam L, Ben Ouezdou M, Sfar Felfoul H, El Mensi R. Characterization of the Tunisian phosphogypsum and its valorization in clay bricks. Construction and Building Materials. 2009; 23: 3240–3247.

[16] El Afifi EM, Hilal MA, Attallah MF, El-Reefy SA. Characterization of phosphogypsum wastes associated with phosphoric acid and fertilizers production. Journal of Environmental Radioactivity. 2009; 100: 407–412. doi: 10.1016/j.jenvrad.2009.01.005 19272681 10.1016/j.jenvrad.2009.01.005

[17] Tayibi H, Choura M, López FA, Alguacil FJ, López-Delgado A. Environmental impact and management of phosphogypsum. Journal of Environmental Management. 2009; 90: 2377–2386. doi: 10.1016/j.jenvman.2009.03.007 19406560 10.1016/j.jenvman.2009.03.007

[18] Ajmal PY, Bhangare RC, Tiwari M, Sahu SK, Pandit GG. External gamma radiation levels and natural radioactivity in soil around a phosphate fertilizer plant at Mumbai. Journal of Radioanalytical and Nuclear Chemistry. 2014; 300(1): 1–5.

[19] Darmoul B, Hadj Ali Salem M, Vitiello P. Effets des rejets industriels de la région de Gabès (Tunisie) sur le milieu marin récepteur. Bulletin de l'Institut National Scientifique et Technique d'Océanographie et de Pêche de Salammbo. 1980; 7: 5–61.

[20] Nafeh Kassir L, Lartiges B, Ouaini N. Effects of fertilizer industry emissions on local soil contamination: a case study of a phosphate plant on the east Mediterranean coast. Environmental Technology. 2012; 33(8): 873–885. 22720412 10.1080/09593330.2011.601765

[21] Rekik A, Drira Z, Guermazi W, Elloumi J, Maalej S, Aleya L et al Impacts of an uncontrolled phosphogypsum dumpsite on summer distribution of phytoplankton, copepods and ciliates in relation to abiotic variables along the near-shore of the southwestern Mediterranean coast. Marine Pollution Bulletin. 2012; 64: 336–346. doi: 10.1016/j.marpolbul.2011.11.005 22154276 10.1016/j.marpolbul.2011.11.005

[22] Wali A, Colinet G, Khadhraoui M, Ksibi M. Trace Metals in Surface Soil Contaminated by Release of Phosphate Industry in the Surroundings of Sfax-Tunisia. Environmental Research, Engineering and Management. 2013; 65(3): 20–30.

[23] Dahri N, Atoui A, Abida H. Environmental Impact Assessment of a Flood Control Channel in Sfax City, Tunisia. International Journal of Science and Engineering. 2014; 7(1): 23–29.

[24] Ayadi N, Aloulou F, Bouzid J. Assessment of contaminated sediment by phosphate fertilizer industrial waste using pollution indices and statistical techniques in the Gulf of Gabes (Tunisia). Arabian Journal of Geosciences. 2014; 8(3): 1–13.

[25] Rabaoui L, Balti R, El Zrelli R, Tlig-Zouari S. Assessment of heavy metal pollution in the gulf of Gabes (Tunisia) using four mollusc species. Mediterranean Marine Science. 2014; 15(1): 45–58.

[26] El Zrelli R, Courjault-Radé P, Rabaoui L, Castet S, Michel S., Bejaoui N. Heavy metal contamination and ecological risk assessment in the surface sediments of the coastal area surrounding the industrial complex of Gabes city, Gulf of Gabes, SE Tunisia. Marine Pollution Bulletin. 2015; 101: 922–929. doi: 10.1016/j.marpolbul.2015.10.047 26526855 10.1016/j.marpolbul.2015.10.047

[27] Ayadi N, Zghal I, Aloulou F, Bouzid J. Impacts of several pollutants on the distribution of recent benthic foraminifera: the southern coast of Gulf of Gabes, Tunisia. Environmental Science and Pollution Research. 2016; 23: 6414–6429. doi: 10.1007/s11356-015-5872-x 26620866 10.1007/s11356-015-5872-x

[28] El Kateb A, Stalder C, Neururer C, Pisapia C, Spezzaferri S. Correlation between pollution and decline of Scleractinian *Cladocora caespitosa* (Linnaeus, 1758) in the Gulf of Gabes. Heliyon. 2016; e00195 doi: 10.1016/j.heliyon.2016.e00195 27896319 10.1016/j.heliyon.2016.e00195 PMC5121140

[29] Ruttenberg KC. Development of a sequential extraction method for different forms of phosphorus in marine sediments. Limnology and Oceanography. 1992; 37(7): 1460–1482.

[30] Berner RA, Ruttenberg KC, Rao JL. The nature of phosphorus burial in modern marine sediments. In: Wollast R., Mackenzie FT, Chou L, editors. Interactions of C, N, P and S: biogeochemical cycles and global change, 4; 1993. pp. 365–378.

[31] Ruttenberg KC, Ogawa NO, Tamburini F, Briggs RA, Colasacco ND, Joyce E. Improved, high-throughput approach for phosphorus speciation in natural sediments via the SEDEX sequential extraction method. Limnology and Oceanography: Methods. 2009; 7: 319–333.

[32] Delaney ML. Phosphorus accumulation in marine sediments and the oceanic phosphorus cycle. Global Biochemical Cycles. 1998; 12(4): 563–572.

[33] Benitez-Nelson CR. The biogeochemical cycling of phosphorus in marine systems. Earth-Science Reviews. 2000; 51: 109–135.

[34] Ruttenberg KC. The global phosphorus cycle. In: Schlesinger WH, editor. Treatise on Geochemistry, 8; 2003. pp. 585–643.

[35] Zaaboub N, Ounis A, Helali MA, Béjaoui B, Lillebø AI, Ferreira da Silva E et al Phosphorus speciation in sediments and assessment of nutrient exchange at the water-sediment interface in a Mediterranean lagoon: Implications for management and restoration. Ecological Engineering. 2014; 73: 115–125.

[36] Cloern JE. Phytoplankton bloom dynamics in coastal ecosystems: A review with some general lessons from sustained investigation of San Francisco Bay, California. Reviews of Geophysics. 1996; 32(2): 127–168.

[37] Correll DL. The role of Phosphorus in the Eutrophication of Receiving Waters: A Review. Journal of Environmental Quality. 1998; 27: 261–266.

[38] Aloulou F., EllEuch B, Kallel M. Benthic foraminiferal assemblages as pollution proxies in the northern coast of Gabes Gulf, Tunisia. Environmental Monitoring and Assessment. 2012; 184: 777–795. doi: 10.1007/s10661-011-2001-2 21476104 10.1007/s10661-011-2001-2

[39] Ben Brahim M, Hamza A, Hannachi I, Rebai A, Jarboui O, Bouain A et al Variability in the structure of epiphytic assemblages of *Posidonia oceanica* in relation to human interferences in the Gulf of Gabes, Tunisia. Marine Environmental Research. 2010; 70: 411–421. doi: 10.1016/j.marenvres.2010.08.005 20851462 10.1016/j.marenvres.2010.08.005

[40] Zaouali J. Les peuplements benthiques de la petite Syrte, golfe de Gabès—Tunisie. Résultats de la campagne de prospection du mois de juillet 1990. Etude préliminaire: biocénoses et thanatocénoses récentes. Marine Life. 1993; 3(1–2): 47–60.

[41] Behar F, Beaumont V, Penteado HL De B. Rock-Eval 6 Technology: Performances and Developments. Oil & Gas Science and Technology—Revue d'IFP Energies nouvelles. 2001; 56(2): 111–134.

[42] Espitalié J, Deroo G, Marquis F. La pyrolyse Rock-Eval et ses applications, deuxième partie. Revue de l’Institut français du pétrole. 1985; 40(6): 755–784.

[43] Lafargue E, Marquis F, Pillot D. Rock-Eval 6 apllication in hydrocarbon exploration, production, and soil contamination studies. Revue de l’Institut français du pétrole. 1998; 53(4): 421–437.

[44] Langford FF, Blanc-Valleron MM. Interpreting Rock-Eval Pyrolysis Data Using Graphs of Pyrolizable Hydrocarbons vs. Total Organic Carbon. The American Association of Petroleum Geologists Bulletin. 1990; 74(6): 799–804.

[45] Katz BJ. Limitations of “Rock-Eval” pyrolysis for typing organic matter. Organic Geochemistry. 1983; 4(3/4): 195–199.

[46] Delvaux D, Martin H, Leplat P, Paulet J. Geochemical characterization of sedimentary organic matter by means of pyrolysis kinetic parameters. Organic Geochemistry. 1990; 16(1–3): 175–187.

[47] Schlitzer R. Ocean Data View, odv.awi.de, 2016.

[48] Lenzi M, Gennaro P, Volterrani M, Roffilli R, Birardi F, Micarelli P et al Human impact on a small barrier reef meadow of *Posidonia oceanica* (L.) Delile on the north Tyrrhenian coast (Italy). Marine Pollution Bulletin. 2013; 77(1–2): 45–54. doi: 10.1016/j.marpolbul.2013.10.036 24229783 10.1016/j.marpolbul.2013.10.036

[49] Nixon SW. Coastal Marine eutrophication: a definition, social causes, and future concerns. OPHELIA. 1995; 41: 199–219.

[50] Smith VH, Joye SB, Howarth RW. Eutrophication of freshwater and marine ecosystems. Limnology and Oceanography. 2006; 51(1): 351–355.

[51] Smith VH, Tilman GD, Nekola JC. Eutrophication: impacts of excess nutrient inputs on freshwater, marine, and terrestrial ecosystems. Environmental Pollution. 1999; 100: 179–196. 15093117 10.1016/s0269-7491(99)00091-3

[52] Smith VH. Eutrophication of Freshwater and Coastal Marine Ecosystems, A Global Problem. Environmental Science and Pollution Research. 2003; 10(2): 126–139. 12729046 10.1065/espr2002.12.142

[53] El Zrelli R, Courjault-Radé P, Rabaoui P, Daghbouj N, Mansour L, Balti R et al Biomonitoring of coastal pollution in the Gulf of Gabes (SE, Tunisia): use of *Posidonia oceanica* seagrass as a bioindicator and its mat as an archive of coastal metallic contamination. Environmental Science and Pollution Research. 2017; 24(28): 22214–22225. doi: 10.1007/s11356-017-9856-x 28795327 10.1007/s11356-017-9856-x

[54] El Lakhrach H, Hattour A, Jarboui O, Elhasni K, Ramos-Espla AA. Spatial distribution and abundance of the megabenthic fauna community in Gabes gulf (Tunisia, eastern Mediterranean Sea). Mediterranean Marine Science. 2012; 13(1): 12–29.

[55] Darmoul B. Pollution dans le Golfe de Gabès (Tunisie) Bilan de six années de surveillance (1976–1981). Bulletin de l'Institut National Scientifique et Technique d'Océanographie et de Pêche de Salammbô. 1988; 15: 61–84.

[56] Hrs-Brenko M. The basket shell, *Corbula gibba* Olivi, 1792 (Bivalve Mollusks) as a species resistant to environmental disturbances: A review. Acta Adriatica. 2006; 47(1): 49–64.

[57] Messaoudi I, Deli T, Kessabi K, Barhoumi S, Kerkeni A, Saïd K. Association of spinal deformities with heavy metal bioaccumulation in natural populations of grass goby, *Zosterisessor ophiocephalus* Pallas, 1811 from the Gulf of Gabès (Tunisia). Environmental Monitoring and Assessment. 2009; 156: 551–560. doi: 10.1007/s10661-008-0504-2 18709433 10.1007/s10661-008-0504-2

[58] Gargouri D, Azri C, Serbaji MM, Jedoui Y, Montacer M. Heavy metal concentrations in the surface marine sediments of Sfax Coast, Tunisia. Environmental Monitoring and Assessment. 2011; 174: 519–530. 10.1007/s10661-010-1548-7 20533086

[59] Haridasan PP, Maniyan CG, Pillai PMB, Khan AH. Dissolution characteristics of ^226^Ra from phosphogypsum. Journal of Environmental Radioactivity. 2002; 62: 287–294. 12164633 10.1016/s0265-931x(02)00011-5

[60] Guo T, Malone RF, Rush KA. Stabilized Phosphogypsum: Class C Fly Ash: Portland Type II Cement Composites for Potential Marine Application. Environmental Science & Technology. 2001; 35(19): 3967–3973. 11642462 10.1021/es010520+

[61] Anderson RF. Chemical tracers of particle transport Treatise on Geochemistry, 6 Oxford, Pergamon; 2003.

[62] Orr JC, Fabry VJ, Aumont O, Bopp L, Doney SC, Feely RA et al Anthropogenic ocean acidification over the twenty-first century and its impact on calcifying organisms. Nature. 2005; 437(7059): 681–686. doi: 10.1038/nature04095 16193043 10.1038/nature04095

[63] Morse JW, Mackenzie FT. Geochemistry of sedimentary carbonates, 48 Elsevier; 1990.

[64] Zeebe RE, Wolf-Gladrow DA. CO_2_ in seawater: equilibrium, kinetics, isotopes, 65 Gulf Professional Publishing; 2001.

[65] Bathurst RGC. Boring algae, micrite envelopes and lithification of molluscan biosparites. Geological Journal. 1966; 5(1): 15–32.

[66] Bathurst RGC. Carbonate sediments and their diagenesis, 12 Elsevier; 1972.

[67] Tucker M E, Bathurst R G C. Carbonate diagenesis–Reprint Series Volume 1 of the International Association of Sedimentologists. Blackwell Scientific Publications; 1990.

[68] Cotner JB, Ammerman JW, Peele ER, Bentzen E. Phosphorus-limited bacterioplankton growth in the Sargasso Sea. Aquatic Microbial Ecology. 1997; 13: 141–149.

[69] McKenzie JA, Vasconcelos C. Dolomite Mountains and the origin of the dolomite rock of which they mainly consist: historical developments and new perspectives. Sedimentology. 2009; 56: 205–219.

[70] Vasconcelos C, McKenzie J, Bernasconi S, Grujic D, Tien AJ. Microbial mediation as a possible mechanism for natural dolomite formation at low temperatures. Nature: 1995; 377: 220–222.

[71] Bontognali TRR, Vasconcelos C, Warthmann RJ, Bernasconi SM, Dupraz C, Strohmenger CJ et al Dolomite formation within microbial mats in the coastal sabkha of Abu Dhabi (United Arab Emirates). Sedimentology. 2010; 57: 824–844.

[72] M’Rabet A. Differentiation of environments of dolomite formation, Lower Cretaceous of Central Tunisia. Sedimentology. 1981; 28: 331–352.

[73] Castany G. Carte géologique de la Tunisie. Sheet N° 1 & 2. 1:500000. Direction des travaux publics service des mines de l’industrie et de l’énergie, Tunis; 1951.

[74] Laffite R, Castany G, Lelubre M. Carte Géologique du Nord-Ouest de l’Afrique Algérie-Tunisie. Sheet N°2. 1:2000000. Comité d’organisation du XIXe Congrès Géologique International, Alger; 1952.

[75] Snelgrove PVR. The Importance of Marine Sediment Biodiversity in Ecosystem Processes. Ambio. 1997; 26(8): 578–583.

[76] Pearson TH, Rosenberg R. Macrobenthic succession in relation to organic enrichment and pollution of the marine environment. Oceanography and Marine Biolology–An Annual Review. 1978; 16: 229–311.

[77] Milliman JD. Organic matter content in US Atlantic continental slope sediments: decoupling the grain-size factor. Deep Sea Research Part II: Topical Studies in Oceanography. 1994; 41(4–6): 797–808.

[78] Secrieru D, Oaie G. The Relation between the Grain Size Composition of the Sediments from the NW Black Sea and their Total Organic Carbon (TOC) Content. Geo-Eco-Marina. 2009; 15: 5–11.

[79] Ben Amor R, Brahim M, Gueddari M. Essai d’interpretation de la dynamique sedimentaire par l’analyse granulometrique et mineralogique au large du Golfe de Gabes. Bulletin de l’Institut National des Sciences et Technologies de la Mer de Salammbô. 2003; 30: 143–151.

[80] Littke R. Deposition, Diagenesis and Weathering of Organic Matter-Rich Sediments Lecture Notes in Earth Sciences,47 Springer Heidelberg, 1993.

[81] Boughzala K, Fattah N, Bouzouita K, Ben Hassine H. Etude minéralogique et chimique du phosphate naturel d’Oum El Khecheb (Gafsa, Tunisie). Revue science des matériaux, Laboratoire LARHYSS. 2015; 6: 11–29.

[82] Nuňez-Betelu L, Baceta JI. Basics and Application of Rock-Eval/TOC Pyrolysis: an example from the uppermost Paleocene/lowermost Eocene In The Basque Basin, Western Pyrenees. MUNIBE (Ciencias Naturales—Natur Zientziak). 1994; 46: 43–62.

[83] Khiari R, Mhenni MF, Belgacem MN, Mauret E. Chemical composition and pulping of date palm rachis and *Posidonia oceanica*–A comparison with other wood and non-wood fibre sources. Bioresource Technology. 2010; 101: 775–780. doi: 10.1016/j.biortech.2009.08.079 19766481 10.1016/j.biortech.2009.08.079

[84] Ben Hassen A, Trichet J, Disnar JR, Belyaouni H. Données nouvelles sur le contenu organique des dépôts phosphatés du gisment de Ras-Draâ (Tunisie) New data on the organic matter associated to phosphatic ores of the Ras-Draâ deposit (Tunisia). Comptes Rendus Géoscience. 2009; 341(4): 319–326.

[85] Dean WE, Arthur MA, Claypool GE. Depletion of ^13^C in cretaceous marine organic matter: Source, diagenetic, or environmental signal? Marine Geology. 1986; 70: 119–157.

[86] Henchiri M. Sedimentation, depositional environment and diagenesis of Eocene biosiliceous deposits in Gafsa basin (southern Tunisia). Journal of African Earth Sciences. 2007; 49: 187–200.

[87] Hayes JM, Strauss H, Kaufman AJ. The abundance of ^13^C in marine organic matter and isotopic fractionation in the global biogeochemical cycle of carbon during the past 800 Ma. Chemical Geology. 1999; 161: 103–125.

[88] Freudenthal T, Wagner T, Wenzhöfer F, Zabel M, Wefer G. Early diagenesis of organic matter from sediments of the eastern subtropical Atlantic: Evidence from stable nitrogen and carbon isotopes. Geochimica et Cosmochimica Acta. 2001; 65(11): 1795–1808.

[89] Patience RL, Clayton CJ, Kearseley AT, Rowland SJ, Bishop AN, Rees AWG et al An integrated biochemical, geochemical, and sedimentological study of organic diagenesis in sediments from LEG 112. Proceedings of the Ocean Drilling Program: Scientific Results, 112 1990: 135–146.

[90] Müller A, Mathesius U. The palaeoenvironments of coastal lagoons in the southern Baltic Sea, I. The application of sedimentary C_org_/N ratios as source indicators of organic matter. Palaeogeography, Palaeoclimatology, Palaeoecology. 1999; 145(1): 1–6.

[91] Bordovskiy OK. Accumulation of organic matter in bottom sediments. Marine Geology. 1965; 3: 33–82.

[92] Gearing JN, Gearing PJ, Rudnick DT, Requejo AG, Hutchins MJ. Isotopic variability of organic carbon in a phytoplankton-based, temperate estuary. Geochimica et Cosmochimica Acta. 1984; 48(5): 1089–1098.

[93] Khan NS, Vane CH, Horton BP. Stable carbon isotope and C/N geochemistry of coastal wetland sediments as a sea level indicator In: Shennan I, Long AJ, Horton BP, editors. Handbook of sea-level research; 1991 pp. 295–311.

[94] Thornton SF, McManus J. Application of Organic Carbon and Nitrogen Stable Isotope and C/N Ratios as Source Indicators of Organic Matter Provenance in Estuarine Systems: Evidence from the Tay Estuary, Scotland. Estuarine, Coastal and Shelf Science. 1994; 38: 219–233.

[95] Hedges JI, Keil RG, Benner R. What happens to terrestrial organic matter in the ocean? Organic Geochemistry. 1997; 27(5/6): 195–212.

[96] Berland BR, Bonin DJ, Maestrini SY. Azote ou phosphore? Considérations sur le “paradoxe nutritionnel” de la mer méditerranée. Oceanologica Acta. 1980; 3(1): 135–142.

[97] De la Rocha CL, Passow U (2003). The biological Pump. In: Elderfield H, editor. Treatise on Geochemistry, 8; 2003. pp. 1–29.

[98] Turner RE, Rabalais NN, Justic D, Dortch Q. Future aquatic nutrient limitations. Marine Pollution Bulletin. 2003; 46: 1032–1034. doi: 10.1016/S0025-326X(03)00049-3 12907197 10.1016/S0025-326X(03)00049-3

[99] Feki W, Hamza A, Bel Hassen M, Rebai A. Les efflorescences phytoplanctoniques dans le Golfe de Gabes (Tunisie) au cours de dix ans de surveillance (1995–2005). Bulletin de l’Institut National des Sciences et Technologies de la Mer de Salammbô. 2008; 35: 105–116.

[100] Smith BN, Epstein S. Two Categories of ^13^C/^12^C Ratios for Higher Plants. Plant Physiology. 1971; 47: 380–384. 16657626 10.1104/pp.47.3.380 PMC365873

[101] Cooper LW, Deniro MJ. Stable carbon isotope variability in the seagrass *Posidonia oceanica*: evidence for light intensity effects. Marine Ecology Progress Series. 1989; 50: 225–229.

[102] Vizzini S, Sarà G, Michener RH, Mazzola A. The role and contribution of the seagrass *Posidonia oceanica* (L.) Delile organic matter for secondary consumers as revealed by carbon and nitrogen stable isotope analysis. Acta Oecologica. 2002; 23: 277–285.

[103] Vizzini S, Sarà G, Mateo MA, Mazzola A. δ^13^C and δ^15^N variability in *Posidonia oceanica* associated with seasonality and plant fraction. Aquatic Botany. 2003; 76: 195–202.

[104] Lepoint G, Dauby P, Fontaine M, Bouquegneau JM, Bobert S. Carbon and Nitrogen Isotopic Ratios of the Seagrass *Posidonia oceanica*: Depth-related Variations. Botanica Marina. 2003; 46: 555–561.

[105] Garcias-Bonet N, Arrieta JM, Duarte CM, Marbà N. Nitrogen-fixing bacteria in Mediterranean seagrass (*Posidonia oceanica*) roots. Aquatic Botany. 2016; 131: 57–60.

[106] Ben Aoun Z, Farhat F, Chouba L, Hadj Ali MS. Investigation on possible chemical pollution on the Boughrara Lagoon, South of Tunisia, by chemical wastes. Bulletin de l’Institut National des Sciences et Technologies de la Mer de Salammbô. 2007; 34: 119–127.

[107] Bejaoui B, Rais S, Koutitonsky V. Modelisation de la dispersion du phosphogypse dans le Golfe de Gabes. Bulletin de l’Institut National des Sciences et Technologies de la Mer de Salammbô. 2004; 31: 103–109.

[108] Baskerville WH, Cameron FK. Ferric oxide and aqueous sulfuric acid at 25°C. The Journal of Physical Chemistry. 1935; 39(6): 769–780.

[109] Klein C, Dutrow BD, James Dwight K. The 23^rd^ edition of the manual of mineral science John Wiley & Sons, inc.; 2007.

[110] Mcartur JM. Francolite geochemistry—compositional controls during formation, diagenesis, metamorphism and weathering. Geochimica et Cosmochimica Acta. 1985; 49(1), 23–35.

[111] Lucotte M, Mucci A, Hillaire-Marcel C, Tran S. Early diagenetic processes in deep Labrador Sea sediments: reactive and nonreactive iron and phosphorus. Canadian Journal of Earth Sciences. 1994; 31(1): 14–27.

[112] van der Zee C, Slomp CP, van Raaphorst W. Authigenic P formation and reactive P burial in sediments of the Nazaré canyon on the Iberian margin (NE Atlantic). Marine Geology. 2002; 185: 379–392.

[113] Slomp CP, Van der Gaast SJ, Van Raaphorst W. Phosphorus binding by poorly crystalline iron oxides in North Sea sediments. Marine Chemistry. 1996; 52: 55–73.

[114] Slomp CP, Epping EHG, Helder W, Van Raaphorst W. A key role for iron-bound phosphorus in authigenic apatite formation in North Atlantic continental platform sediments. Journal of Marine Research. 1996; 54: 1179–1205.

[115] Ruttenberg KC, Sulak DJ. Sorption and desorption of dissolved organic phosphorus onto iron (oxydr)oxides in seawater. Geochimica et Cosmochimica Acta. 2011; 75: 4095–4112.

[116] Oueslati A. The evolution of low Tunisian coasts in historical times: from progradation to erosion and salinization. Quaternary International. 1995; 29/30: 41–47.

[117] Sheldon RP. Ancient marine phosphorites. Annual Review of Earth and Planetary Sciences. 1981; 9(1), 251–284.

[118] Al-Enezi E, Al-Dousari A, Al-Shammari F. Modeling adsorption of inorganic phosphorus on dust fallout in Kuwait bay. Journal of Engineering Research. 2014; 2(2): 1–14.

[119] Søndergaard M. Seasonal Variation in the Loosely Sorbed Phosphorus Fraction of the Sediment of a Shallow and Hypereutrophic Lake. Environmental Geology and Water Sciences. 1988; 11(1): 115–121.

[120] Peng JF, Wang BZ, Song YH, Yuan P, Liu Z. Adsorption and release of phosphorus in the surface sediment of a wastewater stabilization pond. Ecological Engineering. 2007; 31: 92–97.

[121] Van Cappellen P, Berner RA. Fluorapatite crystal growth from modified seawater solutions. Geochimica et Cosmochimica Acta. 1991; 55: 1219–1234.

[122] Koched W, Hattour A, Alemany F, Garcia A, Said K. Spatial distribution of tuna larvae in the Gulf of Gabes (Eastern Mediterranean) in relation with environmental parameters. Mediterranean Marine Science. 2013; 14(1): 5–14.

[123] Hattab T, Ben Rais Lasram F, Albouy C, Romdhane MS, Jarboui O, Halouani G et al An ecosystem model of an exploited southern Mediterranean shelf region (Gulf of Gabes, Tunisia) and a comparison with other Mediterranean ecosystem model properties. Journal of Marine Systems. 2013; 128: 159–174.

